# Inadequate control of thyroid hormones sensitizes to hepatocarcinogenesis and unhealthy aging

**DOI:** 10.18632/aging.102285

**Published:** 2019-09-13

**Authors:** Livia López-Noriega, Vivian Capilla-González, Nadia Cobo-Vuilleumier, Eugenia Martin-Vazquez, Petra Isabel Lorenzo, Enrique Martinez-Force, Mario Soriano-Navarro, María García-Fernández, Silvana Yanina Romero-Zerbo, Francisco Javier Bermúdez-Silva, Irene Díaz-Contreras, Ana Sánchez-Cuesta, Carlos Santos-Ocaña, Abdelkrim Hmadcha, Bernat Soria, Franz Martín, Benoit Raymond Gauthier, Alejandro Martin-Montalvo

**Affiliations:** 1 Department of Regeneration and Cell Therapy, Andalusian Center for Molecular Biology and Regenerative Medicine-CABIMER, Junta de Andalucia-University of Pablo de Olavide-University of Seville-CSIC, Seville, Spain; 2 Instituto de la Grasa (CSIC), Universidad Pablo de Olavide, Sevilla, Spain; 3 Centro de Investigación Príncipe Felipe, Valencia, Spain; 4 Department of Human Physiology, Málaga University, Biomedical Research Institute of Málaga (IBIMA), Málaga, Spain; 5 Instituto de Investigación Biomédica de Málaga-IBIMA, UGC Endocrinología y Nutrición. Hospital Regional Universitario de Málaga, Málaga, Spain; 6 Biomedical Research Network on Diabetes and Related Metabolic Diseases-CIBERDEM, Instituto de Salud Carlos III, Madrid, Spain; 7 Centro Andaluz de Biología del Desarrollo, Universidad Pablo de Olavide and CIBERER, Sevilla, Spain; 8 Deptartment of Physiology, University Miguel Hernández School of Medicine Sant Joan d'Alacant, Alicante, Spain

**Keywords:** lifespan, healthspan, thyroid hormones, hyperthyroidism, hypothyroidism, glucose metabolism

## Abstract

An inverse correlation between thyroid hormone levels and longevity has been reported in several species and reduced thyroid hormone levels have been proposed as a biomarker for healthy aging and metabolic fitness. However, hypothyroidism is a medical condition associated with compromised health and reduced life expectancy. Herein, we show, using wild-type and the Pax8 ablated model of hypothyroidism in mice, that hyperthyroidism and severe hypothyroidism are associated with an overall unhealthy status and shorter lifespan. Mild hypothyroid Pax8 +/- mice were heavier and displayed insulin resistance, hepatic steatosis and increased prevalence of liver cancer yet had normal lifespan. These pathophysiological conditions were precipitated by hepatic mitochondrial dysfunction and oxidative damage accumulation. These findings indicate that individuals carrying mutations on PAX8 may be susceptible to develop liver cancer and/or diabetes and raise concerns regarding the development of interventions aiming to modulate thyroid hormones to promote healthy aging or lifespan in mammals.

## INTRODUCTION

The increasing burden of age-related diseases highlights the importance of uncovering the mechanisms underlying the aging process. Studies in animal models have identified several endocrine and metabolic factors that interact with aging pathways, including insulin, growth hormone (GH), insulin-like growth factor 1 and thyroid hormone (TH) signalling among others [1–3]. The main THs produced in the thyroid gland are triiodothyronine (T3) and thyroxin (T4). In humans, 80% of the THs secreted by the thyroid gland is T4 and 20% is T3. T4 is converted by deiodinases into T3 within cells. TH secretion is tightly regulated under physiological conditions by the hypothalamus-pituitary-thyroid (HPT)-axis. The hypothalamus secretes thyrotropin-releasing hormone (TRH) that induces the transcription of genes required to generate and secrete the thyroid-stimulating hormone (TSH) in the hypophysis. TSH stimulates the production of T4 and T3 in the thyroid gland, which in turn inhibit both TRH and TSH synthesis when THs reach the hypothalamus and the hypophysis, respectively. THs in circulation reach the majority of somatic cells, *via* TH receptors, and modulate the expression of more than 80 genes, such as uncoupling proteins (UCPs), which ultimately produce an increase on the basal metabolic rate [4].

Greater life expectancy has been associated with reduced circulating levels of T4, T3, and/or high TSH levels in both, animal models and humans [3, 5–8]. In this line, the Laron (GH Receptor Knock Out; Ghr KO), Ames (Prop1-mutated) and Snell (Pit1-mutated) dwarf mice, which have reduced GH signalling and reduced circulating TH levels, exhibit a consistent exceptional lifespan as well as other metabolic alterations such as enhanced hepatic insulin sensitivity [9–12]. Both, rodents and humans under calorie restriction (CR), which comprises a variety of nutritional interventions with several beneficial effects including extended longevity, exhibit reduced circulating T3 levels and/or high TSH levels [13–15]. Interestingly, CR mimetics such as resveratrol alter the HPT axis (*e.g.* elevating TSH levels), suggesting that the modulation of THs might contribute to the beneficial effects conferred by these interventions [16].

Studies in humans have revealed that centenarians and their offspring display higher TSH levels in blood [17]. Likewise, nonagenarians from families with exceptional long lifespans, as well as their descendants, have been reported to exhibit increased TSH levels and/or decreased circulating T3 levels [18, 19]. Actually, mildly down-regulated thyroid function has been suggested to correlate with better function in old age and it has been proposed as a biomarker of healthy aging [18, 20, 21].

Several reports indicate that THs induce reactive oxygen species (ROS) production and oxidative stress, which could provide a causal link with aging [22–24]. However, THs have been shown to promote mitophagy and upregulate the expression of *Ucp2*, which might reduce ROS production [25–27]. In addition, epidemiological observations have indicated that, both hypothyroidism and subclinical hypothyroidism, are serious medical conditions associated with cardiovascular problems and several metabolic disorders, such as steatosis and cancer, that reduce health and increase mortality risk [28–31].

These contradictory observations led us to assess the specific effect of the modulation of TH levels in murine healthspan and lifespan using the Pax8 knock-out animal model of hypothyroidism (Wild type [Wt], Pax8 +/- and Pax8 -/-) supplemented or not with THs.

## RESULTS

### High or low TH levels compromise murine healthspan and lifespan

**Figure 1:**
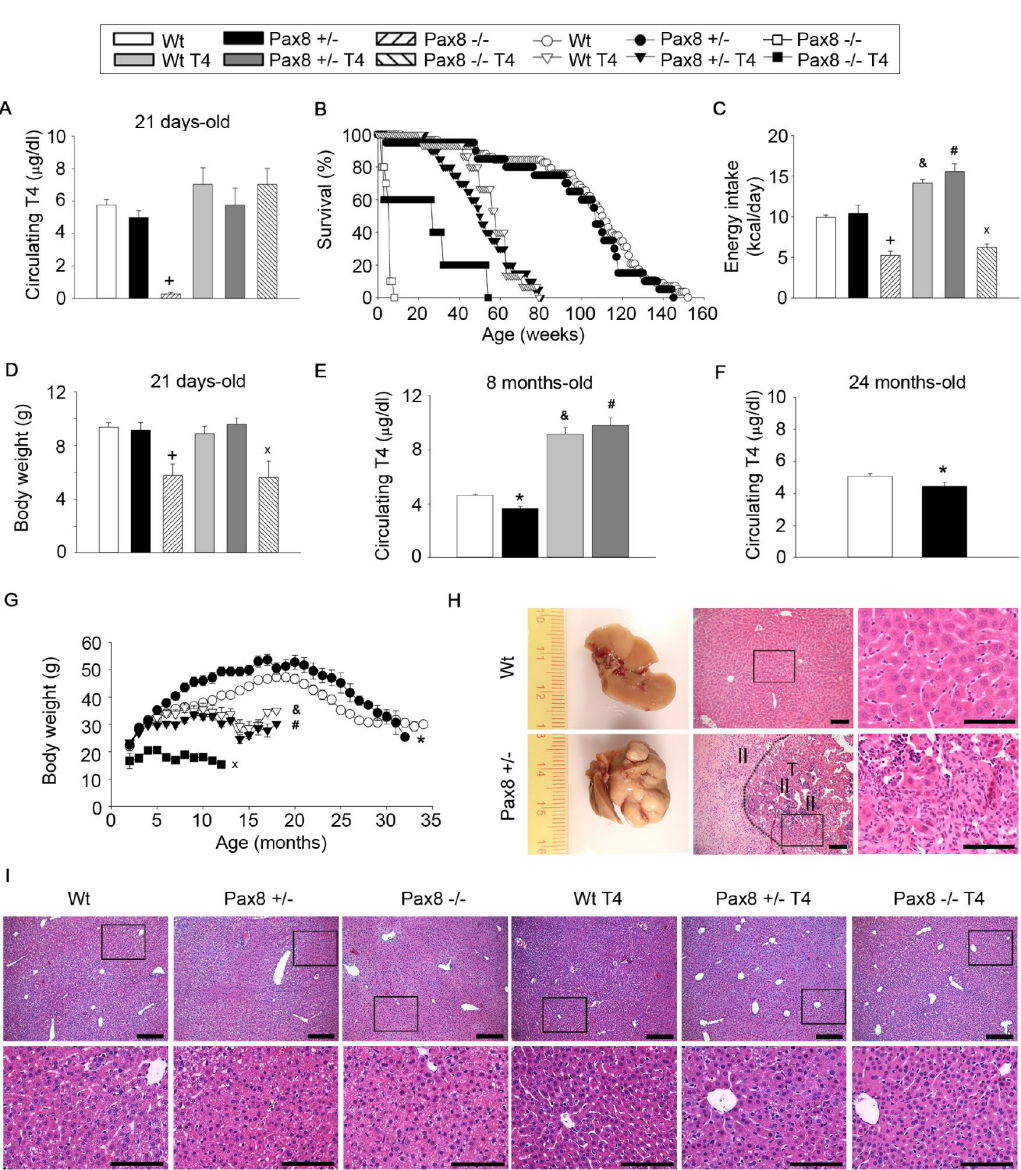
**High or low TH levels compromise murine healthspan and lifespan.** (**A**) Circulating T4 levels at 21 days of age. n = 5 Wt, n = 5 Pax8 +/-, n = 5 Pax8 -/-, n = 5 Wt T4, n = 5 Pax8 +/- T4, n = 3 Pax8 -/- T4. Two-way ANOVA. (**B**) Kaplan-Meier survival curve. n = 58 Wt, n = 20 Pax8 +/-, n = 10 Pax8 -/-, n = 15 Wt T4, n = 20 Pax8 +/- T4, n = 5 Pax8 -/- T4. Survival log rank test. (**C**) Basal daily energy intake in 5-weeks old mice. n = 5 Wt, n = 4 Pax8 +/-, n = 3 Pax8 -/-, n= 3 Wt T4, n = 3 Pax8 +/- T4, n = 3 Pax8 -/- T4 (the “n” reflects the number of cages). Two-way ANOVA. (**D**) Body weight at 21 days of age. n = 21 Wt, n = 15 Pax8 +/-, n = 5 Pax8 -/-, n = 11 Wt T4, n = 11 Pax8 +/- T4, n = 4 Pax8 -/- T4. Two-way ANOVA. (**E**) Circulating T4 levels at 8 months of age. n = 11 Wt, n = 5 Pax8 +/-, n = 7 Wt T4, n = 5 Pax8 +/- T4. Two-way ANOVA. (**F**) Circulating T4 levels at 24 months of age. n = 8 per group. T-test two tailed. (**G**) Body weight during longevity assay. n = all available mice of longevity assay (panel B). One-way ANOVA or ANOVA on ranks if distribution is not normal. * on months 6-17 and 21-28. & on months 12-18. # on months 3, 5-8 and 10-18. x on months 3-12. (**H**) Representative photographs and hematoxylin and eosin staining of liver tissue from Wt and Pax8 +/- mice at necropsies. Wt liver exhibits normal hepatic parenchyma with acinar architecture. Pax8 +/- liver displays primary liver epithelial neoplasms (T) with disorganized architecture and inflammatory infiltrate (II). Scale bar 100 um. n = 4 per group. See also Supplementary Figure 1K. (**I**) Histological analysis of liver tissue by hematoxylin and eosin staining at 21 days of age. n = 4 Wt, n = 5 Pax8 +/-, n = 5 Pax8 -/-, n = 5 Wt T4, n = 5 Pax8 +/- T4, n = 3 Pax8 -/- T4. Scale bars; top: 200 μm; down: 100 μm. Data are represented as the mean ± SEM. * p-value < 0.05 between Wt mice and Pax8 +/- mice. + p-value < 0.05 between Wt mice and Pax8 -/- mice. & p-value < 0.05 between Wt mice and Wt T4 mice. # p-value < 0.05 between Wt mice and Pax8 +/- T4 mice. x p-value < 0.05 between Wt mice and Pax8 -/- T4.

In order to determine the effect of lower levels of THs in mouse healthspan and lifespan, we used the murine Pax8 model of hypothyroidism [32]. In adulthood, *Pax8* is predominantly expressed in kidney and thyroid, while absent in several metabolic tissues (Supplementary Figure 1A). Pax8 -/- mice at 21 days of age were almost devoid of *Pax8* expression in the thyroid as well as circulating T4 levels, while maintaining normal α-glycoprotein subunit (α-GSU) levels of gonadotropic hormones when compared to Wt mice (Figure 1A and Supplementary Figure 1B–1C). Pax8 -/- mice displayed lower body and tissue weights, reduced food intake and hypoglycemia that result in peri-weaning death (χ^2^ ≥ 100 and *P* < 0.0001 vs. Wt) (Figure 1B–1D and Supplementary Figure 1D–1G). *Pax8* expression in the thyroid as well as circulating T4 levels at weaning (21 days) were similar in Pax8 +/- mice when compared to Wt mice, whereas α-GSU was increased (Figure 1A and Supplementary Figure 1B–1C). As such, energy intake as well as body, organ weights and glycaemia were similar at 21 days of life in Wt and Pax8 +/- mice (Figure 1C–1D and Supplementary Figure 1D–1G). However, by the age of 8 months, *Pax8* expression was decreased in Pax8 +/- mice when compared to Wt mice, resulting in a mild but significant reduction in T4 circulating levels, an effect maintained for up to 24 months (Figure 1E–1F and Supplementary Figure 1H). Levels of α-GSU were similar to Wt levels at these time points (Supplementary Figure 1I–1J). Lower circulating T4 levels correlated with increased body weight in Pax8 +/- mice as compared to Wt mice (Figure 1G). Although not significant, Pax8 +/- mice showed a trend towards a shorter life expectancy when compared to Wt mice (Figure 1B). Necropsy revealed that Pax8 +/- mice were susceptible to develop with age liver cancers with hallmarks of hepatocellular carcinoma, as no gross anatomical alterations were detected at day 21 of life (Figure 1H–1I, Supplementary Figure 1K and Supplementary Table 1). Taken together these data suggest that severe loss of TH in Pax8-/- mice is lethal while modest reduction observed in Pax8 +/- mice is associated with altered metabolism as assessed by increased susceptibility to obesity and liver cancer.

We next assessed the impact of TH supplementation on health and longevity by treating or not, Wt, Pax8 +/- and Pax8 -/- male mice with T4. No differences were detected in circulating T4 levels in 21-day old T4-treated Wt (Wt T4) and T4-treated Pax8 +/- (Pax8 +/- T4) mice, which also exhibited normal liver histology (Figure 1A and 1I). At 8-month of age T4 levels were approximately 2-fold higher in Wt T4 and Pax8 +/- T4 mice as compared to Wt mice (Figure 1E). Although, energy intake was increased in both groups, Wt T4 and Pax8 +/- T4 mice remained significantly lighter than their untreated counterparts (Figure 1C and 1G). Lifespan was reduced in Wt T4 and Pax8 +/- T4 mice (Figure 1B). Lifespan was also shortened in T4-treated Pax8 -/- (Pax8 -/- T4) when compared to Wt mice (Figure 1B). Although T4 levels were similar to Wt mice in 21 days-old Pax8 -/- T4 mice, energy intake, body and organs weight was decreased in these mice (Figure 1A, 1D and Supplementary Figure 1D–1G). These data reveal that T4 supplementation in Wt and Pax8 deficient mice compromises lifespan.

### The modulation of THs alters glucose homeostasis in adulthood

**Figure 2:**
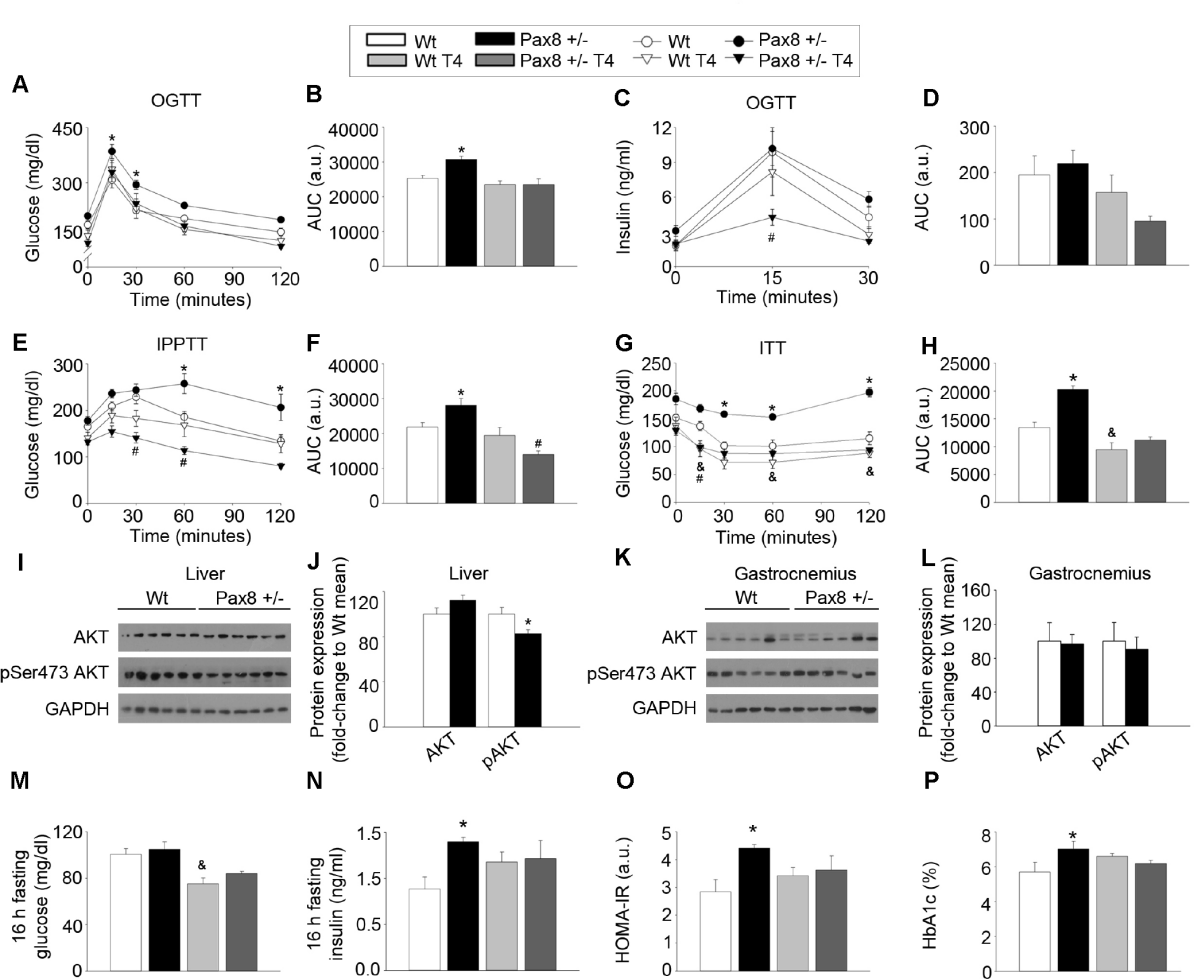
**The modulation of THs alters glucose homeostasis in adulthood.** (**A**) Circulating glucose levels during an OGTT. n = 8 per group. Repeated measurements two-way ANOVA. See also Supplementary Figure 2A. (**B**) AUC for the OGTT. Two-way ANOVA. (**C**) Circulating insulin levels during an OGTT. n = 8 per group. Repeated measurements two-way ANOVA. See also Supplementary Figure 2B. (**D**) AUC for the insulin levels in the OGTT. Two-way ANOVA. (**E**) Circulating glucose levels during an IPPTT. n = 8 per group. Repeated measurements two-way ANOVA. See also Supplementary Figure 2C. (**F**) AUC for the IPPTT. Two-way ANOVA. (**G**) Circulating glucose levels during an ITT. n = 8 per group. Repeated measurements two-way ANOVA. See also Supplementary Figure 2D. (**H**) AUC for the ITT. Two-way ANOVA. (**I**) Western blots showing total Akt and pSer473 Akt in liver extracts of Wt and Pax8 +/- mice. Mice were 9 month-old at time of death and were fasted 16 hours prior killing. (**J**) Densitometric analysis of western blots shown in panel I. n = 6 per group. T-test two tailed. (**K**) Western blots showing total Akt and pSer473 Akt in gastrocnemius extracts of Wt and Pax8 +/- mice. Mice were 9 month-old at time of death and were fasted 16 hours prior killing. (**L**) Densitometric analysis of western blots shown in panel K. n = 6 per group. T-test two tailed. (**M**) Glucose levels in blood after 16 hours of fasting. n = 8 per group. Two-way ANOVA. (**N**) Circulating insulin levels after 16 hours of fasting. n = 8 per group. Two-way ANOVA. (**O**) HOMA-IR. n= 8 per group. Two-way ANOVA. (**P**) Percentage of HbA1c. n = 7 per group. Two-way ANOVA. Data are represented as the mean ± SEM. Unless otherwise stated, mice were 7-month old at the time of experimentation. h: hours. a.u.: arbitrary units. * p-value < 0.05 between Wt mice and Pax8 +/- mice. & p-value < 0.05 between Wt mice and Wt T4 mice. # p-value < 0.05 between Wt mice and Pax8 +/- T4 mice.

We recently reported that loss-of-function mutations in the human *PAX8* gene are associated with gestational diabetes mellitus and potentially with type 2 diabetes mellitus in males [33, 34]. As these mutations are also linked to hypothyroidism, we determined whether glucose metabolism was affected in adult (~7-month old) male Pax8 mice and whether T4 treatment could alleviate symptoms. Of note, Pax8 -/- and Pax8 -/- T4 mice were not included in these analyses due to their limited survival. Pax8 +/- mice exhibited mild glucose intolerance as compared to Wt mice even when values were corrected for basal glucose levels (Figure 2A–2B and Supplementary Figure 2A). Insulin levels during an oral glucose tolerance test (OGTT) were similar in Pax8 +/- mice, suggesting that glucose intolerance in these animals is not due to major defects in glucose-stimulated insulin secretion (Figure 2C–2D and Supplementary Figure 2B). We next performed an intraperitoneal (ip) pyruvate tolerance test (IPPTT) to investigate potential alterations in hepatic gluconeogenesis. Pax8 +/- mice exhibited increased glucose levels in blood compared to Wt mice at 60 and 120 minutes after the pyruvate load which led to an increase in the area under the curve (AUC) (Figure 2E–2F and Supplementary Figure 2C). Pax8 +/- mice displayed severe insulin resistance, as determined by increased AUC during the insulin tolerance test (ITT) (Figure 2G–2H and Supplementary Figure 2D). Confirming the insulin resistance observed in metabolic tests, western blots performed in liver extracts revealed a decrease in pSer473 Akt in Pax8 +/- mice (Figure 2I–2J). In contrast, pSer473 Akt levels were similar in gastrocnemius extracts of Pax8 +/- mice (Figure 2K–2L). Pax8 +/- mice exhibited an increased homeostatic assessment of insulin resistance (HOMA-IR) index, which was caused by fasting hyperinsulinemia (Figure 2M–2O). Pax8 +/- male mice also exhibited increased levels of glycated hemoglobin (HbA1c) (Figure 2P). Therefore, these results suggest that insulin resistance in Pax8 +/- mice is mainly due to impaired hepatic insulin sensitivity.

Wt T4 male mice exhibited restricted levels of circulating glucose during the ITT as well as in fasting conditions, as previously described in Wt T4-treated female mice (Figure 2G, H, M) [35]. Noticeably, the majority of metabolic tests indicated that T4 supplementation in Pax8 +/- mice normalized the metabolic phenotype of mild hypothyroid Pax8 +/- mice, suggesting that restricted TH levels in Pax8 +/- mice are responsible for the metabolic phenotype (Figure 2A–2H, 2M–2P).

### High Fat Diet (HFD) feeding does not exacerbates the metabolic phenotype of Pax8 +/- mice

**Figure 3:**
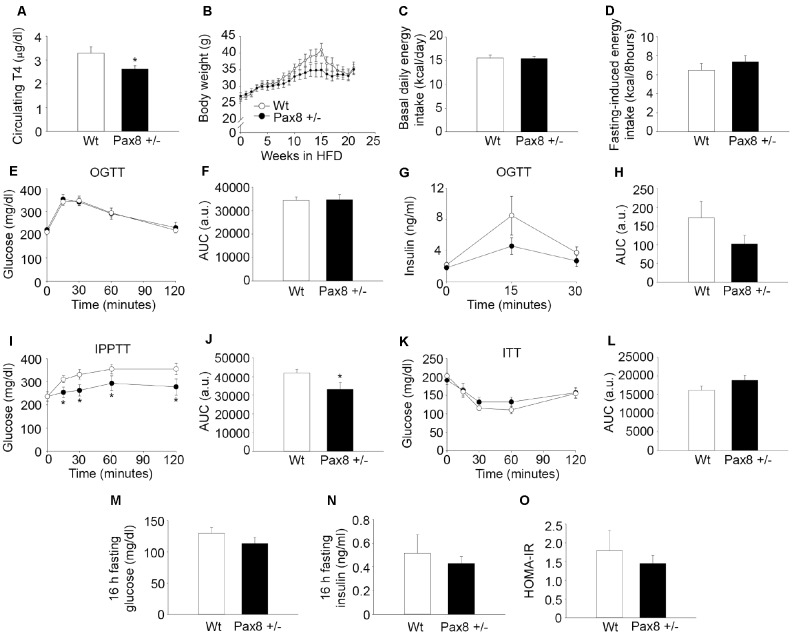
**High fat diet (HFD) feeding does not exacerbates the metabolic phenotype of Pax8 +/- mice.** (**A**) T4 levels in serum (18 weeks of HFD feeding). n = 8 Wt, n = 9 Pax8 +/-. T-test two tailed. (**B**) Body weights during HFD feeding. n = 15 Wt, n = 14 Pax8 +/-. Repeated measurements two-way ANOVA. (**C**) Basal daily energy intake at 6 months of life (13 weeks of HFD feeding). n = 8 Wt, n = 9 Pax8 +/-. T-test two tailed. (**D**) Fasting induced energy intake (17 weeks of HFD feeding). n = 8 Wt, n = 9 Pax8 +/-. T-test two tailed. (**E**) Circulating glucose levels during an OGTT at 6 months of life (14 weeks of HFD feeding). n = 13 Wt, n = 11 Pax8 +/-. Repeated measurements two-way ANOVA. (**F**) AUC for glucose levels during the OGTT. T-test two tailed. (**G**) Insulin levels in plasma during an OGTT (17 weeks of HFD feeding). n = 8 per group. Repeated measurements two-way ANOVA. (**H**) AUC for insulin levels during the OGTT. T-test two tailed. (**I**) Circulating glucose levels during the IPPTT (15 weeks of HFD feeding). n = 13 Wt, n = 10 Pax8 +/-. Repeated measurements two-way ANOVA. (**J**) AUC for the IPPTT. T-test two tailed. (**K**) Glucose levels in blood during the ITT (16 weeks of HFD feeding). n = 9 per group. Repeated measurements two-way ANOVA. (**L**) AUC for the ITT. T-test two tailed. (**M**) Glucose levels in blood after 16 hours of fasting (18 weeks of HFD feeding). n = 8 per group. T-test two tailed. (**N**) Circulating insulin after 16 hours of fasting (18 weeks of HFD feeding). n = 8 per group. T-test two tailed. (**O**) HOMA-IR (18 weeks of HFD feeding). n = 8 per group. T-test two tailed. Data are represented as the mean ± SEM. * p-value <0.05. Unless otherwise stated, mice were 7-month old at the time of experimentation.

In order to investigate whether the phenotype observed in Pax8 +/- mice was further exacerbated by a metabolic challenge, we determined the effects of HFD feeding in adult Pax8 +/- mice. Metabolic parameters were assessed in ~7 month-old mice that had been on a HFD for up to 18 weeks in order to compare and contrast with experiments performed in standard diet-fed (STD) mice (Figures 1 and 2). Circulating T4 levels were slightly lower in HFD-fed Pax8 +/- mice when compared to HFD-fed Wt mice, while α-GSU levels remained similar (Figure 3A and Supplementary Figure 3A). Weight gain and energy intake were similar in HFD-fed Wt and HFD-fed Pax8 +/- mice (Figure 3B–3D). As expected, HFD-fed Wt mice exhibited compromised glucose clearance when compared to STD-fed Wt mice (Figures 2A–2B and 3E–3F). Glucose levels during an OGTT were similar between the different genotypes in HFD and comparable to STD-fed Pax8 +/- mice (Figure 2A–2B and 3E–3F). We observed a tendency towards decreased levels of circulating insulin at 15 minutes after a glucose load in HFD-fed Pax8 +/- mice (Figure 3G–3H). . Glucose levels were lower in HFD-fed Pax8 +/- mice at all time-points during an IPPTT when compared to HFD-fed Wt mice, suggesting compromised pyruvate-induced hepatic gluconeogenesis (Figure 3I–3J). In contrast, nonsignificant differences were observed between the different genotypes fed on HFD in an ITT, fasting blood glucose, fasting circulating insulin, HOMA-IR index, spatial learning and memory as well as in the weight of the main metabolic tissues at the time of sacrifice (Figure 3K–3O and Supplementary Figure 3B–3I). Overall these results indicate that Pax8 +/- mice suffer metabolic complications, irrespective of the energetic content of the diet.

### Mild hypothyroid Pax8 +/- mice exhibit reduced basal metabolic rate and compromised physical performance

**Figure 4:**
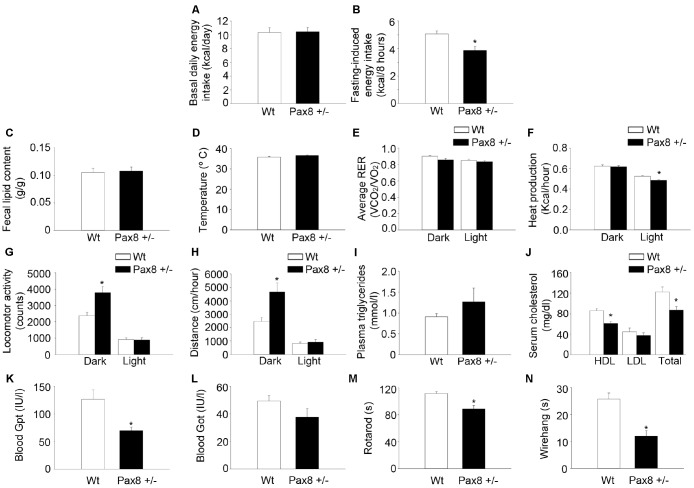
**Mild hypothyroid Pax8 +/- mice exhibit reduced basal metabolic rate and compromised physical performance.** (**A**) Basal daily energy intake per day at 10-12 months of life. n = 5 Wt, n = 4 Pax8 +/- (the n reflects the number of cages). (**B**) Fasting-induced energy intake at 10-12 months of life. n = 21 Wt, n = 14 Pax8 +/-. (**C**) Lipid content in faeces. n = 7 Wt, n = 6 Pax8 +/-. (**D**) Body temperature. n = 12 Wt, n = 8 Pax8 +/-. (**E**) Determination of RER in metabolic cages. n = 8 Wt, n = 7 Pax8 +/-. (**F**) Determination of heat production in metabolic cages. n = 8 Wt, n = 7 Pax8 +/-. (**G**) Determination of spontaneous locomotor activity (y + x axis) in metabolic cages. n = 8 Wt, n = 7 Pax8 +/-. (**F**) Determination of run distance in metabolic cages. n = 8 Wt, n = 7 Pax8 +/-. (**H**) Triglyceride concentration in plasma. n = 18 Wt, n = 13 Pax8 +/-. (**I**) Total cholesterol, HDL and LDL/VLDL levels in serum. n = 7 per group. (**J**) Blood levels of Gpt. n = 7 per group. (**K**) Blood levels of Got. n = 7 per group. (**L**) Time to fall from an accelerating rotarod at 7-10 months of life. n = 27 Wt, n = 20 Pax8 +/-. (**M**) Time to fall in wire hang test at 7-10 months of life. n = 27 Wt, n = 20 Pax8 +/-. Data are represented as the mean ± SEM. Unless otherwise stated, mice were 7-month old at the time of experimentation. * p-value < 0.05. T-test two tailed.

In order to further characterize the metabolic alterations associated with mild hypothyroidism in adult Pax8 +/- mice, we assessed several physiological parameters. Despite having increased body weight (Figure 1G), adult Pax8 +/- mice showed similar daily energy intake, water intake and reduced fasting-induced energy intake when compared to Wt mice (Figure 4A–4B and Supplementary Figure 4A–4B). No differences were observed in either fecal lipid content or body temperature (Figure 4C–4D). *In vivo* indirect calorimetry indicated that the respiratory exchange ratio (RER) was unaffected (Figure 4E). In contrast, heat production was decreased in Pax8 +/- mice specifically during light-time, a time at which mice are less active (Figure 4F–4H). During dark-time Pax8 +/- mice exhibited a greater spontaneous locomotor activity and ran longer distances when compared to their Wt counterparts (Figure 4G–4H). Heat production was similar between different genotypes at that specific part of the day cycle (Figure 4F). The analysis of circulating lipids indicated that total cholesterol levels were lower in Pax8 +/- mice as a result of reduced HDL with no changes in triglyceride levels (Figure 4I–4J). Lower levels of HDL may stem from liver dysfunction/damage. Nonetheless, blood levels of glutamic-pyruvic transaminase (Gpt) and glutamic oxaloacetic transaminase (Got) were lower and unaltered, respectively, in Pax8 +/- mice as compared to Wt mice indicating normal liver function (Figure 4K–4L). Mild hypothyroid Pax8 +/- mice performed poorly on rotarod and wire hang physical tests (Figure 4M–4N). Histological analyses of gastrocnemius by optic microscopy and electron microscopy indicated similar proportion of type I and type II myofibers, as well as comparable morphology and ultrastructure of subsarcolemmal and intermyofibrillar mitochondrial populations, suggesting that compromised physical activity is likely caused by the increased body weight rather than detrimental functional effects in skeletal muscle (Supplementary Figure 4C–4F). Finally, Pax8 +/- and Wt mice shared identical spatial learning and memory, indicating no alterations in neurocognitive function (Supplementary Figure 4G–4L). These results indicate that Pax8 +/- mild hypothyroid male mice exhibit an overall unhealthy status which involves a lower basal metabolic rate, a clinical feature associated with human hypothyroidism [36].

### Mild hypothyroid Pax8 +/- mice accumulate ectopic lipid depositions

**Figure 5:**
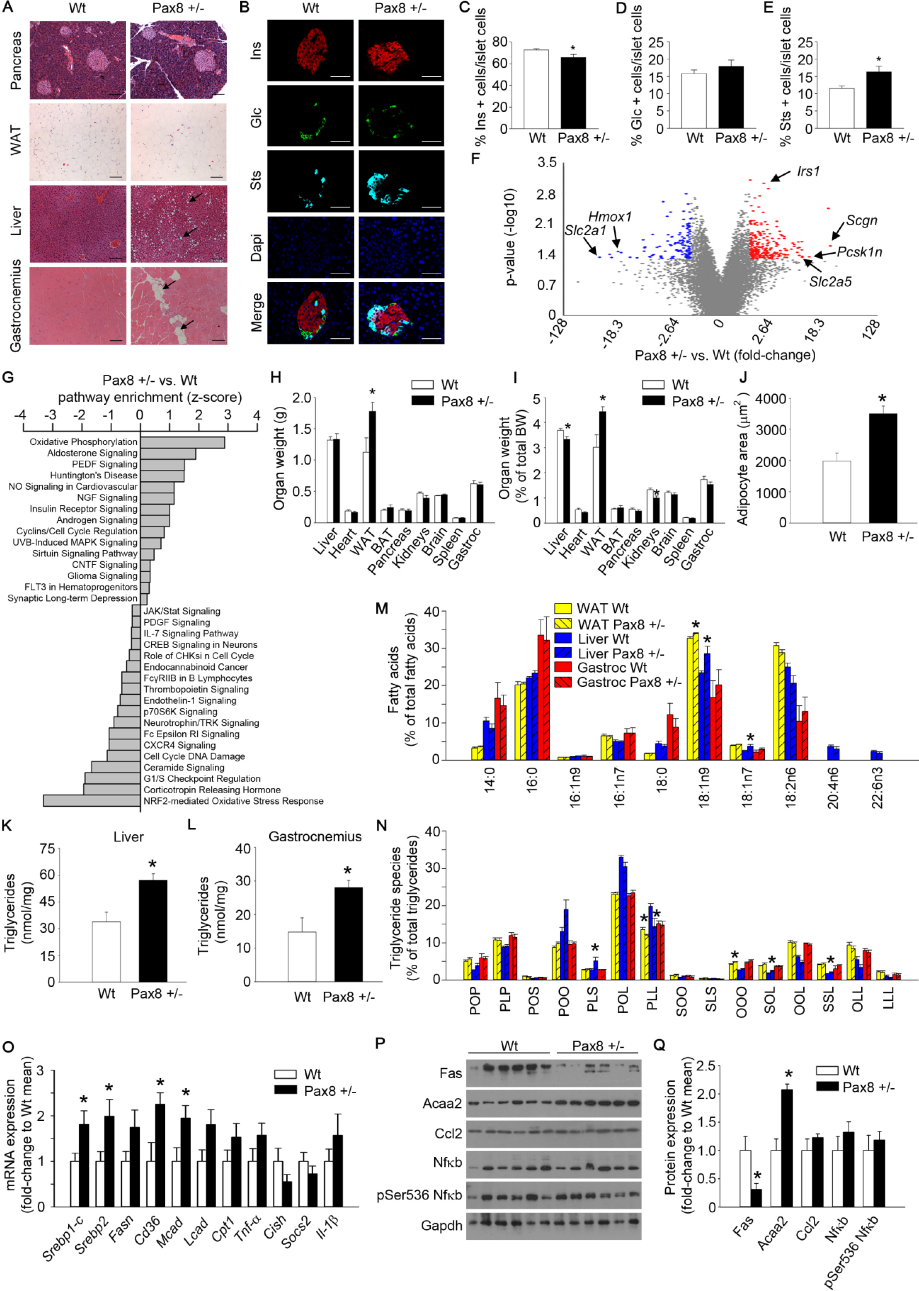
**Mild hypothyroid Pax8 +/- mice accumulate ectopic lipid depositions.** (**A**) Representative images of hematoxylin and eosin staining of WAT, gastrocnemius, pancreas and liver of Wt and Pax8 +/- mice. Arrows indicate adipocyte infiltrations in gastrocnemius and lipid droplets in liver tissue. WAT: n = 6 Wt, n = 4 Pax8 +/-; gastrocnemius: n = 6 per group; pancreas: n = 3 per group; liver: n = 6 per group; Scale bar = 100 μm. (**B**) Representative immunofluorescence images of pancreatic sections from untreated Wt and Pax8 +/- male mice at 9 months of age. Scale bar: 50 μm. Ins: insulin. Glc: glucagon. Sts: somatostatin. n = 5 Wt, n = 4 Pax8 +/-. (**C**) Percentage of insulin positive cells in islet cells at 9 months of age. n = 5 Wt, n = 4 Pax8 +/-. T-test two tailed. (**D**) Percentage of glucagon positive cells in islet cells at 9 months of age. n = 5 Wt, n = 4 Pax8 +/-. T-test two tailed. (**E**) Percentage of somatostatin positive cells in islet cells at 9 months of age. n = 5 Wt, n = 4 Pax8 +/-. T-test two tailed. (**F**) Volcano plot showing the fold change and statistical significance of genes expressed in pancreatic islets. Statistical analysis was performed using Transcription Analysis Console using default parameters. Arrows indicate specific dots of highlighted genes. See also Supplementary Figure 5E–5G. n = 3 per group. (**G**) Analysis of significantly modulated annotated canonical pathways in pancreatic islets using Ingenuity Pathway Analysis platform. Statistical analysis was performed using Ingenuity Pathway Analysis using significantly modulated genes. n = 3 per group. (**H**) Tissues weight. n = 6 per group. T-test two tailed. (**I**) Tissues weight corrected by total body weight. n = 6 per group. T-test two tailed. (**J**) Quantification of adipocyte area. n = 6 Wt, n = 4 Pax8 +/-. T-test two tailed. (**K**) Triglyceride content per mg of protein in liver. n = 6 Wt, n = 4 Pax8 +/-. T-test two tailed. (**L**) Triglyceride content per mg of protein in gastrocnemius. n = 5 Wt, n = 4 Pax8 +/-. T-test two tailed. (**M**) Lipidomic analysis depicting percentages of the different species of fatty acids in WAT, liver and gastrocnemius. n = 5 Wt, n = 6 Pax8 +/-. Two-way ANOVA. See also Supplementary Figure 5H. (**N**) Lipidomic analysis depicting percentages of triglyceride species in WAT, liver and gastrocnemius. P: Palmitic acid. O: Oleic acid. L: Linoleic acid. S: Stearic acid. n = 5 Wt, n = 6 Pax8 +/-. Two-way ANOVA. (**O**) mRNA expression of genes involved in lipid synthesis/import and inflammation in livers from Wt and Pax8 +/- mice. *Srebp1-c*: n = 6 per group. *Srebp2*: n = 6 per group. *Fasn*: n = 5 Wt, n = 6 Pax8 +/-. *Cd36*: n = 6 Wt, n = 5 Pax8 +/-. *Mcad*: n = 6 per group. *Lcad*: n = 6 per group. *Cpt1*: n = 6 per group. *Tnf-α*: n = 5 Wt, n = 6 Pax8 +/-. *Cish*: n = 4 Wt, n = 5 Pax8 +/-. *Socs2*: n = 4 Wt, n = 4 Pax8 +/-. *Il-1β*: n = 6 per group. T-test two tailed. (**P**) Western blots showing protein expression levels of markers of lipid metabolism and inflammation in liver lysates. n = 6 per group. (**Q**) Densitometric analysis of western blots shown in panel P. n = 6 per group. T-test two tailed. Data are represented as the mean ± SEM. Mice were 9 month-old at the time of killing. vs: versus. * p-value < 0.05.

Histological analysis of the four predominant organs involved in metabolic homeostasis revealed larger adipocytes and aberrant lipid accumulation in the liver and gastrocnemius of 9-month old Pax8 +/- mice (Figure 5A). Although pancreatic islet architecture appeared normal without evidence of lipid accumulation and insulin levels were not altered in adult Pax8 +/- mice during an OGTT (Figure 2C–2D and Figure 5A), at 9-month old Pax8 +/- mice exhibited a reduced percentage of insulin-expressing pancreatic β cells and a concomitant increase in somatostatin-expressing pancreatic δ-cells (Figure 5B–5E). These differences were not observed at weaning (21 days-old mice) (Supplementary Figure 5A–5D). To better understand the impact of mild hypothyroidism on pancreatic islets, we performed a genome-wide transcriptomic analysis. The expression of 232 genes was upregulated (fold-change ≥ 2.5; p value ≤ 0.05) while the expression of 119 genes was downregulated in islets of Pax8 +/- mice (fold-change ≤-2.5; p value ≤ 0.05). Among the most up-regulated genes were secretagogin (*scgn*), the proprotein convertase Subtilisin/Kexin Type 1 Inhibitor (*Pcsk1n*) and the solute carrier family 2 member 5, also known as glucose transporter 5 (*Slc2a5*/*Glut5*), which encode for proteins involved in β-cell turnover, glucose uptake (preferentially in α-cells) and insulin processing and secretion (Figure 5F and Supplementary Table 2) [37, 38]. Top significantly downregulated genes included genes encoding for the glucose transporter 1 (*Slc2a1*/*Glut1*), and the Heme-oxigenase1 (*Hmox1*), which encode for glucose transporters and antioxidant proteins, respectively (Figure 5F and Supplementary Table 2). Analyses using the Ingenuity Pathway Analysis and Transcriptome Analysis Console platforms for genome-wide expression analyses indicated that oxidative phosphorylation (up-regulated) and antioxidant stress response (down-regulated) were among the most modulated pathways in pancreatic islets isolated from mild hypothyroid Pax8 +/- mice (Figure 5G and Supplementary Figure 5E–5G).

Consistent with lipid accumulation, tissue weight at sacrifice (9-month old) indicated that the white adipose tissue (WAT) of Pax8 +/- mice was heavier, even when corrected by total body weight, and was composed of larger adipocytes when compared to the WAT of Wt mice (Figure 5A, 5H–5J). Furthermore, liver and gastrocnemius of Pax8 +/- mice accumulated greater amounts of triglycerides (Figure 5K–5L). Given that THs are potent modulators of fatty acid composition and storage, and that aberrant accumulation of certain lipid species lead to cell dysfunction and death (lipotoxicity), we performed lipidomic analysis on WAT, liver and gastrocnemius [39]. The analysis of WAT indicated that mild hypothyroid Pax8 +/- mice accumulated a higher percentage of oleic acid (18:1n9) as well as increased proportion of triglycerides formed by 3 oleic acids (OOO), with a concomitant reduction of the proportion of triglycerides formed by other fatty acids such as palmitic acid and linolenic acid (PLL) (Figure 5M–5N). However, the percentage of different polar lipid species was comparable to Wt mice, suggesting the absence of major alterations in cellular membrane integrity or fluidity (Supplementary Figure 5H). Similarly, the percentage of 18:1n9, as well as 18:1n7, fatty acid species was higher in the liver of Pax8 +/- mice as compared to Wt mice, while no significant differences were observed in the gastrocnemius (Figure 5M). The analysis of triglyceride species in liver lysates indicated a reduction of certain triglycerides composed by more than one poli-unsaturated fatty acid and a concomitant increase in triglycerides composed by saturated fatty acids, such as stearic acid (Figure 5N). No differences in proportions of polar lipid species were found in liver and gastrocnemius tissues (Supplementary Figure 5H). RNA and protein isolations of liver tissue exhibited a marked modulation on genes and proteins involved in lipid transport, synthesis and catabolism (Figure 5O–5Q). Transcript levels of *Srebp-1c*, *Srebp2* which are involved in fatty acid and cholesterol biosynthesis, respectively, were induced in the liver but not in the gastrocnemii (*Srebp-1c*) of Pax8 +/- mice (Figure 5O and Supplementary Figure 5I). Although the liver of Pax8 +/- mice exhibited more lipid droplets, the expression level of fatty acid synthase (Fas) was restricted, suggesting that free fatty acid supplies for liver triglyceride synthesis are likely provided by circulating lipoproteins (Figure 5P–5Q). Consistent with this premise, expression of the fatty acid scavenger receptor *Cd36* was significantly increased in liver of Pax8 +/- mice (Figure 5O). In parallel, RNA expression levels of the medium chain acyl-CoA dehydrogenase (*Mcad*) and protein expression levels of acetyl-coenzyme A acyltransferase 2 (Acaa2), which catalyze essential steps on the mitochondrial β-oxidation of fatty acids, were markedly increased in the livers of Pax8 +/- mice compared to Wt, suggesting greater hepatic beta oxidation (Figure 5O–5Q). No significant differences were observed in mRNA and/or protein levels of pro-inflammatory markers (Figure 5O–5Q). None of the above alterations were apparent in the gastrocnemius (Supplementary Figure 5I–5K). These results indicate that mild hypothyroidism in Pax8 +/- mice does not result in an inflammatory milieu, which is commonly found in individuals suffering metabolic disorders [40].

### Mild hypothyroidism produces mitochondrial dysfunction and accumulation of oxidative damage in Pax8 +/- mice

**Figure 6:**
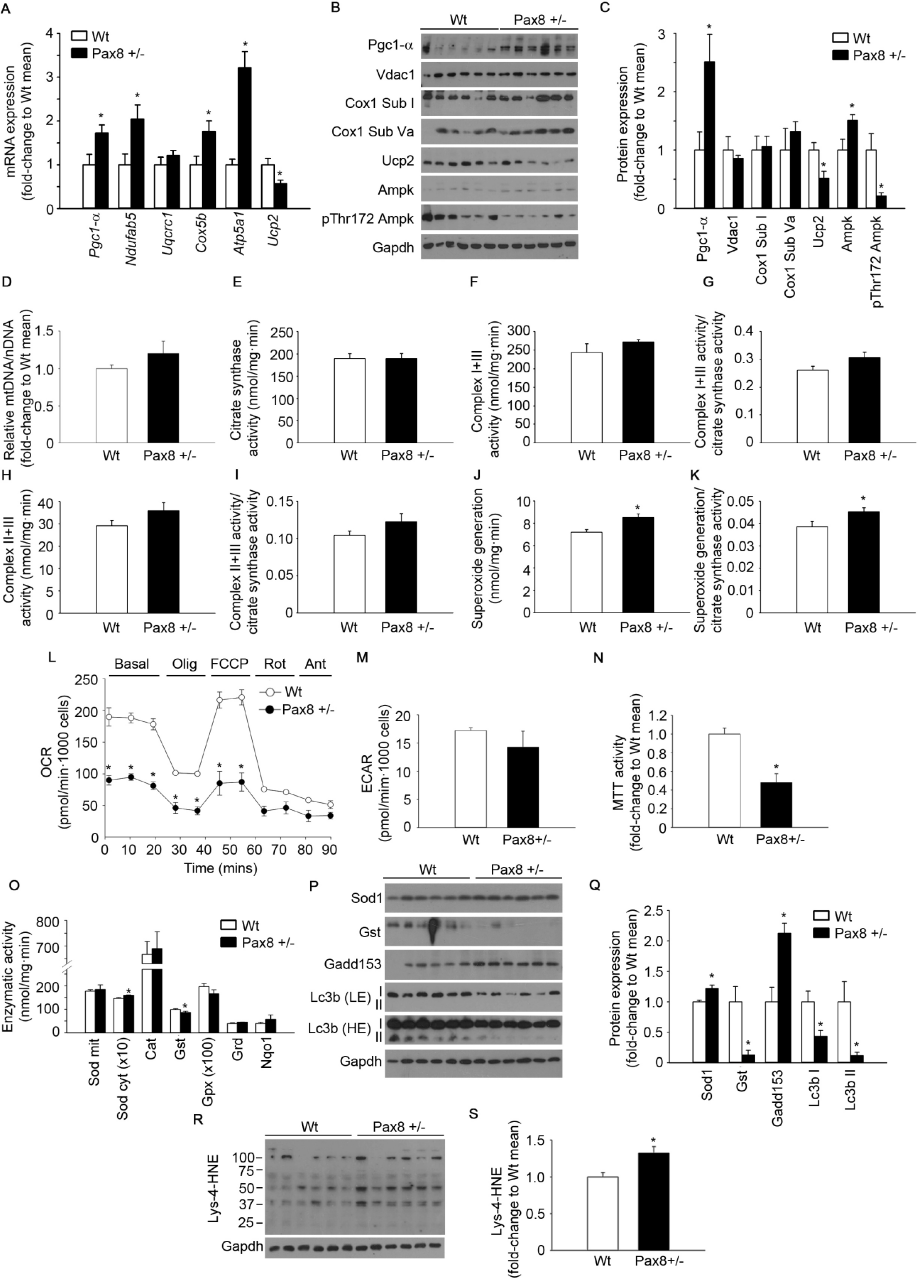
**Mild hypothyroidism produces mitochondrial dysfunction and accumulation of oxidative damage in Pax8 +/- mice.** (**A**) mRNA expression of genes involved in mitochondrial biogenesis and mitochondrial function in liver. *Pgc1-α*: n = 6 per group. *Ndufab5* = n = 6 per group. *Uqcrc1*: n = 6 per group. *Cox5b*: n = 6 per group. *Atp5a1*: n = 5 Wt, n = 6 Pax8 +/-. *Ucp2*: n = 5 per group. (**B**) Western blots showing expression levels of proteins involved in mitochondrial biogenesis/function as well as Ampk and its phosphorylated isoform in liver lysates. n = 6 per group. (**C**) Densitometric analysis of western blots shown in panel B. n = 6 per group. (**D**) Relative mitochondrial DNA content in liver isolations. n = 6 per group. (**E**) Citrate synthase activity in liver extracts. n = 6 per group. (**F**) Complex I+III activity in liver extracts. n = 6 per group. (**G**) Complex I+III activity corrected by citrate synthase activity in liver extracts. n = 6 per group. (**H**) Complex II+III activity in liver extracts. n = 6 per group. (**I**) Complex II+III activity corrected by citrate synthase activity in liver extracts. n = 6 per group. (**J**) Superoxide generation in mitochondrial complexes in liver extracts. n = 6 per group. (**K**) Superoxide generation in mitochondrial complexes corrected by citrate synthase activity in liver extracts. n = 6 per group. (**L**) Oxygen consumption rate in primary hepatocytes. n = 3 per group. (**M**) Extracellular acidification rate in primary hepatocytes. n = 3 per group. (**N**) MTT test to determine the metabolic activity (NADH-oxidase) of primary hepatocytes. n = 4 per group. (**O**) Antioxidant enzymatic activities in liver lysates. Sod mit: n = 6 per group. Sod cyt: n = 5 per group. Cat: n = 5 Wt, n = 6 Pax8 +/-. Gst: n = 5 per group. Gpx: n = 5 per group. Grd: n = 6 Wt, n = 5 Pax8 +/-. Nqo1: n = 5 Wt, n = 6 Pax8 +/-. Sod mit: Sod mitochondrial. Sod cyt: Sod cytosolic. (**P**) Western blots showing expression levels of antioxidant, stress response and autophagy induction proteins in liver lysates. LE = Low exposure. HE = High exposure. n = 6 per group. (**Q**) Densitometric analysis of western blots shown in panel **P**. (**R**) Western blot showing Lys-4-HNE staining in liver extracts. n = 6 per group. (**S**) Densitometric analysis of the western blot shown in panel **R**. Mice were 9 month-old at the time of killing. Data are represented as the mean ± SEM. * p-value < 0.05. T-test two tailed.

Mitochondrial dysfunction and oxidative stress have been detected in patients with non-alcoholic fatty liver disease (NAFLD) [41, 42]. Therefore, we next investigated whether mitochondria content and function were altered in Pax8 +/- mice . Although transcript and protein levels of Pgc1-α, a master regulator of mitochondrial biogenesis, were increased in the liver of Pax8 +/- mice, the expression of several mitochondrial proteins was similar in mild hypothyroid Pax8 +/- mice (Figure 6A–6C). In agreement with similar mitochondrial content, the liver of Pax8 +/- mice displayed similar mitochondrial DNA to nuclear DNA ratio and citrate synthase activity (Figure 6D–6E). Likewise, no differences were detected between genotypes in the specific enzymatic activities of complexes I+III (NADH-cytochrome *c* oxidoreductase activity) and II+III (succinate-cytochrome *c* oxidoreductase activity) in liver lysates, even when corrected to citrate synthase activity (Figure 6F–6I). Interestingly, gene expression levels of *Atp5a1*, a subunit of complex V involved in ATP synthesis, was increased in the liver of Pax8 +/- mice (Figure 6A). We also observed a marked reduction in Ucp2 expression in the liver of Pax8 +/- mice, consistent with this uncoupling protein being a direct target of THs (Figure 6A–6C). Protein levels of pThr172 Ampk were reduced in liver of Pax8 +/- mice compared to Wt, indicative of a rich energetic status in hepatic tissue (Figure 6B–6C). Remarkably, superoxide generation in mitochondrial complexes was significantly increased in the liver of Pax8 +/- animals, even when corrected to citrate synthase activity (Figure 6J–6K). To assess net effects in mitochondrial performance, we determined *in vivo* oxygen consumption in primary hepatocytes isolated from Wt and Pax8 +/- mice. Although the extracellular acidification rate (ECAR) under basal conditions was similar in both experimental groups, oxygen consumption was reduced in Pax8 +/- hepatocytes (Figure 6L–6M). Interestingly, carbonyl cyanide 4-(trifluoromethoxy) phenylhydrazone (FCCP)-induced maximal oxygen consumption was minimal in the hepatocytes of Pax8 +/- mice (Figure 6L). Confirmatory experiments using the 3-(4,5-dimethylthiazol-2-yl)-2,5-diphenyltetrazolium bromide (MTT) test indicated that metabolic activity was reduced in primary hepatocytes isolated from in Pax8 +/- mice (Figure 6N). We next determined the protein levels and enzymatic activities of several antioxidant proteins (Figure 6O–6Q). Results indicated that liver extracts from Pax8 +/- mice exhibited increased cytosolic superoxide dismutase (Sod cyt) and reduced glutathione S transferase (Gst) protein levels and enzymatic activities, suggesting that hepatic tissue of Pax8 +/- mice senses a pro-oxidant status and modulate the expression/activity of certain antioxidant proteins (Figure 6P–6Q). In addition, protein levels of growth arrest DNA damage 153 (Gadd 153) were increased and Lc3b-I and Lc3b-II levels were reduced in liver extracts of Pax8 +/- mice, indicating that hepatic cells are under cellular stress and that autophagy induction may be impaired in the liver of Pax8 +/- mice (Figure 6P–6Q). Consistent with increased ROS generation and altered antioxidant activity, determinations of lysine-4-hydroxinonenal (Lys-4-HNE) levels, which result from lipid peroxidation, revealed a significant increase in the liver of Pax8 +/- mice (Figure 6R–6S). Despite a dramatic increase in Pgc1-α protein expression, no differences were detected in RNA and protein expression levels of mitochondrial genes/proteins including Ucp2 or in respiratory complex activities in gastrocnemius extracts of Pax8 +/- mice (Supplementary Figure 6A–6I). Although mitochondrial ROS generation and antioxidant protein levels were similar in both Pax8 +/- and Wt mice, Lys-4-HNE levels were higher in Pax8 +/- mice (Supplementary Figure 6J–6O). Taken together, these indicate that, in liver tissue, the antioxidant system is unable to scavenge increased ROS production, leading to the accumulation of oxidative damage in a process that is known to be involved in carcinogenesis.

## DISCUSSION

Harnessing mechanisms governing organismic senescence is central to aging biology in order to develop approaches for healthy aging, a priority of current times. Herein, we tackle this problematic by resolving the controversy on whether the modulation of TH levels is beneficial or detrimental for the health and welfare of individuals. Although our study only included male mice, representing a limitation of our work, we provide evidence that either the lack or chronic supplementation of TH reduces life expectancy while mild hypothyroidism compromises healthspan without extending lifespan. Our study also substantiates that patients bearing *PAX8* mutations resulting in mild hypothyroidism, are prone to develop diabetes (type 2 and gestational), as evidenced by our previous study [33], and likely to develop liver cancer. With regard to the latter, both, hypothyroidism and SNP variants in the *PAX8* gene, have been associated with the development of hepatocellular carcinomas in Asian and Non-Hispanic white cohorts [43, 44].

**Figure 7:**
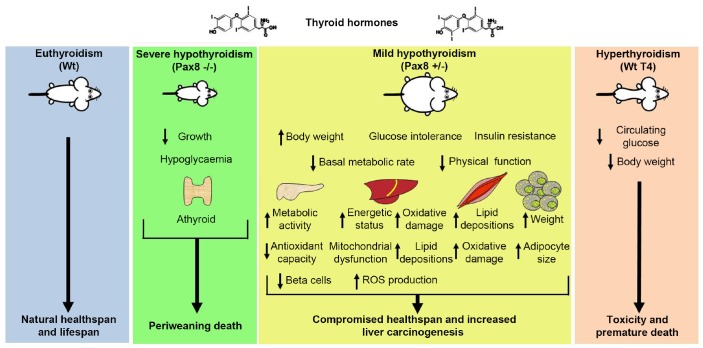
**Schematic representation of the effects of the modulation of THs levels in healthspan and lifespan in mice.** Mice unable to synthesize THs exhibit growth retardation and periweaning death. Mice exhibiting a mild reduction on circulating THs levels develop obesity, glucose metabolism dysregulations and increased incidence of liver cancers, which compromise their quality of life. Mice exhibiting increased levels of THs exhibit reduced body weight and reduced glycaemia at fasting. However, these mice develop toxicity and exhibit a short lifespan. Interventions based on the modulation of THs reduce healthspan and/or lifespan in mammals.

Mice that lack the ability to produce TH exhibited very low body weight and limited survival, dying around weaning (Figure 7). This fact is particularly interesting since the longest-living laboratory mice are characterized by low to barely detectable levels of THs. Our findings indicate that the modulation of other hormones (*e.g.* growth hormones) must be exclusively responsible for the increases in lifespan in other dwarf mice such as the Snell, Ames and Laron mice [5, 7, 9–11]. Our data reveal that T4 supplementation in Pax8 ablated mice extended life expectancy when compared to untreated Pax8 -/- mice while the same treatment reduced by half lifespan in both Wt and Pax8 heterozygous animals. Combined with our previous work [35], our results support that TH supplementation leading to hyperthyroidism produces an improvement on markers of metabolic health, such as reduced age-dependent body weight gain and fasting glycaemia but that chronic treatment results in toxicity and reduced lifespan. These results are consistent with human studies demonstrating that life expectancy for both, men and women, decreased with higher levels of TH [45], and highlights the strict requirement to maintain TH levels within physiological levels during replacement therapy to maintain the health and welfare of patients (Figure 7) [46].

Pax8 heterozygous mice that displayed mild reduction in TH levels did not live longer compared to wild type mice and featured mild obesity, insulin resistance, increased WAT weight, triglyceride accumulation in skeletal muscle, liver steatosis as well as preponderance to develop liver cancer with age. These metabolic alterations recapitulate alterations observed in patients suffering hypothyroidism such as, metabolic syndrome and NAFLD, Type 2 diabetes and hepatocellular carcinomas [28, 33, 43, 44, 47–49]. Therefore, we propose the Pax8 +/- mouse as a valuable experimental model to further dissect the cellular and molecular mechanisms associated with mild hypothyroidism and hypothyroidism-related pathologies. However, Pax8 +/- mice did not exhibit a pro-inflammatory milieu in the liver or the skeletal muscle, which is commonly associated with metabolic disorders [40]. Similar to humans, TH supplementation reversed diseases hallmarks in Pax8 +/- mice, supporting the premise that reduced TH levels rather than *Pax8* haploinsufficiency or direct transcriptional effects of Pax8 targets convey these pathophysiological conditions [47]. Further substantiating this premise is the lack of *Pax8* expression in liver, WAT and skeletal muscle. The phenotype of Pax8 heterozygous mice did not worsen when fed a HFD, indicating that mice are metabolically stressed independent of diet.

Although pancreatic islets appeared architecturally normal at weaning, a reduction in the percentage of beta cell with a concomitant increase in delta cells was observed in adult mice. Whether the modulation of endocrine populations in pancreatic islets in adulthood is compensatory mechanism or whether these are independent cellular events remains to be clarified. Nonetheless, TH facilitate maturation of human fetal islet beta cells as well as neonatal rat beta cells, a process mediated by up-regulation of the beta cell-enriched transcription factor MafA [50, 51]. We also previously demonstrated that TH delayed beta cell loss under stress conditions and that mutations on *PAX8* are found in patients developing gestational diabetes [33, 35]. It is therefore tempting to speculate that hypothyroidism in Pax8 +/- mice hinders postnatal beta cell maturation with subsequent increased in the susceptibility to stress-induced apoptosis with age, favouring the replacement of beta cells by delta cells that do not require MafA. Evidence for stress-induced apoptosis is provided by the transcriptome profile of Pax8 +/- islets that reveal, on one hand, higher metabolism-secretion coupling, likely due to hypothyroidism-induced insulin resistance, and, on the other hand, lower antioxidant defences, likely resulting in β-cell exhaustion and apoptosis at long-term. Further studies are required to assess the impact of hypothyroidism on delta cell fate.

Liver lipidomic profiling revealed oleic acid was enriched in Pax8 +/- mice consistent with a previous study demonstrating that increased intake of this fatty acid promotes hepatoma progression by reducing the expression of Pten [52]. In contrast, other studies have reported that oleic acid is beneficial rather than detrimental due to its capacity to revert palmitic acid-induced hepatic insulin resistance and apoptosis and that the dietary palmitic acid:oleic acid ratio impacts diabetes risk in human [53]. Contextually, hypothyroidism may short-circuit the beneficial role of oleic acid by skewing accumulation of oleic acid and triglycerides, independent of palmitic acid, which long-term may cause NAFLD. Consistent with this premise and in line with results observed in Pax8 +/- mice, oleic acid was markedly increased whereas total serum cholesterol was decreased in mice over-expressing SREBPs, which are downstream targets of the TH signalling pathway [54]. Engorgement of hepatic tissue with lipids was likely the cause of insulin resistance, as evidenced by decreased Akt phosphorylation and contributed to the energy-rich status, as revealed by decreased AMPK phosphorylation. Consistent with this status several markers of mitochondrial beta oxidation were enhanced. Nonetheless, we observed mitochondrial dysfunction as revealed by reduced oxygen consumption in primary live hepatocytes and increased mitochondrial superoxide generation in liver lysates. Of interest, *Cd36* expression, a long-chain fatty acid scavenger receptor found to be increased in the liver of Pax8 +/- mice, is also highly expressed in patients with NAFLD and appears to be required for fatty acid-induced ROS production [55, 56]. In parallel the expression and enzymatic activity of several antioxidant response proteins was altered with a net accumulation of oxidative damage. These data suggests that hepatic tissue senses a pro-oxidant milieu but it is inefficient in scavenging ROS-induced damage, in a process known to be involved in carcinogenesis [57]. In line with the concept that compromised mitochondrial functionality and accumulation of oxidative damage are contributory factors to the development of most age-related diseases, our observations provide direct evidence that subtle reductions on THs, as found in humans with mild hypothyroidism, augment oxidative damage, increase the incidence of liver cancers and reduce healthspan.

In 1908 Dr. Max Rubner proposed the rate of living theory of aging and longevity, postulating that species with a low metabolic rate would have increased life expectancy when compared to species with a higher metabolic rate. In this line, restricted levels of THs, which control the metabolic rate, have been associated with increased longevity as well as metabolic fitness. Therefore, a mildly reduced thyroid axis activity has been proposed as a biomarker for healthy aging in humans [18, 21]. Here, we found that either lack of or high THs levels results in decreased lifespan and/or healthspan whereas mild reduction leads to hallmarks of NAFLD and liver cancer in mice. Our data support the notion that humans with exceptional longevity exhibiting restricted activity of the hypothalamus-pituitary-thyroid axis must have a specific genetic and/or epigenetic signature required to achieve longevity benefits [3, 6, 8]. More importantly, our data indicate that interventions based on the modulation of THs should not be targeted to increase the healthspan and lifespan in healthy mammals including humans.

## METHODS

### Ethics statement

Investigation has been conducted in accordance with the ethical standards and according to the Declaration of Helsinki and according to national and international guidelines and has been approved by the authors´ institutional review board.

### Mice management

Procedures involving the use of live animals were approved by the CABIMER Ethical Committee of Animal Experimentation and performed in accordance with the Spanish law on animal use RD 53/2013 and the EU Directive 2010/63/EU for animal experiments. Mice (*Mus musculus*) were housed in individually ventilated cages (Tecniplast, Buguggiate, Italy) in a specific pathogen-free facility and kept under controlled environmental conditions (12 hours-light–dark cycle, 23 ± 1 °C with 30–50% relative humidity). Souralit plus 29/12 bedding (Souralit, Gerona, Spain) was sterilized by autoclave and added to each cage. Pax8 +/- mice in C57BL/6 background were obtained from Infrafrontiers mouse repository and genotyped according to previously published protocols [32, 58]. Male mice were used in all experiments reported. Mice were provided with standard rodent chow (Envigo, TD2914), unless stated otherwise, and sterilized tap water *ad libitum.* For the HFD study, a chow that provides 60 % of calories from fat was used (Envigo, TD06414) and treatment was started at 2-3 months of age. Mice were treated or not with T4 according to previously published protocols with minor modifications [35, 59, 60]. In brief, T4 treatment was started at birth (subcutaneous injections of 18 ng/g of body weight daily until weaning). After weaning, mice were supplemented with T4 in drinking water (5 μg/ml). Body weights were monitored monthly for the duration of the study. 9 month-old mice were fasted for 16 hours prior killing. 21-days old mice were not fasted prior killing.

### Blinding, group size and randomization

The data analyst was blinded, whereas the operator was not blind to the group assignment of animals. Mice were selected from the pool eligible for inclusion in the study and were randomly divided into the experimental groups according to the genotype. Quantitative analysis of gene and protein expression was normalized to the mean of the control group to facilitate representation and understanding of the results. The experiments shown in Supplementary Figure 2A, 2B, 2C, 2D were normalized to glucose levels at time 0 (%) to determine whether differences in metabolic test stem from differences in basal glucose levels.

### Metabolic tests

For the OGTT, mice were fasted for 6 h at 10 a.m. and received an oral dose of glucose (3 g/kg) by gavage. For the IPPTT, mice were fasted for 6 h from 10 a.m. and received an injection of sodium pyruvate (ip, 2 g/kg) by oral gavage. For the ITT, mice were fasted for 3 h from 10 a.m. and were injected with insulin (ip, 1.5 IU/kg). For the HOMA-IR, mice were fasted from 8 p.m. and glucose and insulin samples were taken at 16 hours of fasting. To determine glucose levels, blood samples were taken by venipuncture using a Precision Xceed glucometer (Abbott, Madrid, Spain). Insulin was measured in plasma using ELISA kits (Crystal Chem, Downers Grove, IL, USA).

### Rotarod

Results from rotarod tests are presented as the time to fall from an accelerating rotarod (4–40 rpm over 5 minutes). Mice were given a 1-minute habituation trial at 4 rpm on the day before the experiment. Results shown are the averages of three trials per mouse [61].

### Wire hang test

For wire hang test, mice were allowed to grip a horizontal 1-mm wire with four paws up to 60 seconds and the latency to fall from the wire was determined. Three different trials were performed with each mouse [61].

### MTT test

Hepatocyte metabolic activity was assessed using the Cell Proliferation Kit I (MTT) according to the manufacturer´s recommendations (Roche, Spain). Optical density was determined at 550 nm with a reference wavelength of 650 nm using a Varioskan Flash spectrophotometer (Thermo Scientific, Spain).

### Transmission electron microscopy

Fresh gastrocnemius muscles were fixed overnight at room temperature in 2.5 % glutaraldehyde and 2% paraformaldehyde in 0.1 M phosphate buffer and then 72 h at 4ºC before processing for electron microscopy. Muscle samples were post-fixed with 2% osmium, rinsed, dehydrated and embedded in Durcupan resin (Fluka, Sigma-Aldrich, St. Louis, USA). To verify orientation, serial semithin sections (1.5 mm) were cut with an Ultracut UC-6 (Leica microsystems, Wetzlar, Germany) and mounted into slides and stained with 1% toluidine blue. In selected correct orientation samples, ultrathin sections (0.07 – 0.08 mm) were cut using an Ultra 45º diamond knife (Diatome Ltd, Nidau, Switzerland) and stained with lead citrate. Finally, photomicrographs were obtained under a transmission electron microscope FEI Tecnai G2 Spirit BioTwin (ThermoFisher Scientific company, Oregon, USA), using a digital camera Morada (Olympus Soft Image Solutions GmbH, Münster, Germany).

### Semi-quantitative RT–PCR

Total RNA was extracted from tissue samples using the Easy-blue RNA extraction kit (Intron Biotechnology, Gyeonggi-doQiagen, Korea). Genomic DNA for mitochondrial DNA determinations, was extracted using the DNeasy Blood & tissue kit (QIAGEN, 60504), according to manufacturer’s protocol. cDNA using 0.5–2 μg RNA was synthesized using SuperScript II Reverse Transcriptase (Thermo Fisher Scientific, Madrid, Spain). Real time PCR was performed on individual cDNAs or genomic DNA using SYBR green (Roche). Primer sequences are presented in Supplementary Table 4. The mRNA expression was calculated by the 2^-ΔΔCT^ method and normalized to the expression of *Rps29*.

### Western blot

Samples were lysed in radioimmunoprecipitation assay buffer (20 mM Tris–HCl (pH 7.5), 150 mM NaCl, 1 mM Na2EDTA, 1 mM EGTA, 1% NP-40, 1% sodium deoxycholate) with protease, phosphatase and deacetylase inhibitors P0044, P5725, P8340 and SC-362323. Western blots were performed according to standard methods, which involved incubation with a primary antibody of interest, followed by incubation with a horseradish peroxidase-conjugated secondary antibody and enhanced chemiluminescence (Supplementary Table 3). Blots were quantified with ImageJ, and the bands of interest were normalized to Ponceau S and/or Gapdh staining, as previously validated [62].

### Islet isolation and transcriptome profiling

Pancreatic islets were isolated using a collagenase perfusion as previously described [33, 63]. cRNA preparations from murine islets were hybridized to mouse Clariom™ S Assay microarray chips, using the standard protocols of the Genomic Core Facility, CABIMER (Affymetrix, Santa CA, USA). Image analysis, quality control and quantification of data were performed using the Affymetrix GeneChip Command Console Software 4.0. Further statistic data analysis was performed using the Transcriptome Analysis Console developed by Affymetrix. Modulated pathways using Transcriptome Analysis Console software were obtained applying significantly modulated genes with a fold-change greater than 2.5. The Ingenuity Pathway Analysis platform was used to determine modulations on annotated “canonical pathways” using significantly modulated genes. Pathway pictures were generated using the annotated “canonical pathways” module of Ingenuity Pathway Analysis. Raw data are accessible at GSE123288.

### Oxygen consumption and ECAR

Mitochondrial bioenergetics of primary hepatocytes cells were measured using an XF24 Extracellular Flux Analyzer (Agilent) [64]. After overnight culture cells were washed with Seahorse assay media (Seahorse Bioscience), supplemented with pyruvate at 1 mM and glutamine at 2 mM. Plates were incubated in a CO_2_-free incubator at 37 °C for 1 h to allow temperature and pH equilibration, after which oxygen consumption rate (OCR) was measured in the XF24 Extracellular Flux Analyzer over a period of 88 min. Mitochondrial processes were examined through sequential injections of oligomycin (4 μM) at min 21, carbonyl cyanide 4-(trifluoromethoxy) phenylhydrazone (FCCP; 2 μM) at min 39, rotenone (1 μM) at min 57, and antimycin A (5 μM) at min 75. ECAR was determined under basal culture conditions. At the end of the measurement, cells in each well were counted using the Scepter™ 2.0 Cell Counter to normalize the data.

### Densitometry quantifications

Western blots and the area of adipocytes were quantified with ImageJ software (NIH). Quantifications of cell number/percentage were performed manually using Adobe Photoshop (Adobe).

### Quantifications and statistical analyses

For comparisons between two groups, statistical significance was calculated using non-paired two-tailed student’s t-test using Excel software. For other comparisons a repeated measurements two-way ANOVA test, one-way ANOVA, ANOVA on ranks or two-way ANOVA were performed using Graph Pad Prism 6 (GraphPad) or SigmaPlot 12.0 (SigmaPlot) software. A log rank statistical test was applied for survival curves (SigmaPlot). Specific statistical tests and the number of biological replicates (n) used in each panel are stated in Figure legends. Errors are represented as the standard error of the mean (SEM). For all analyses, p-value < 0.05 was considered statistically significant. Plots were designed with the software SigmaPlot 12.5 (SigmaPlot).

## Supplementary Materials

Supplementary Methods

Supplementary Figures

Supplementary Tables
