# Supplementary material for: Inadequate control of thyroid hormones sensitizes to hepatocarcinogenesis and unhealthy aging
Source: Aging (Albany NY). 2019 Sep 13;11(18):7746–79. doi: 10.18632/aging.102285 (PMC6781991; doi:10.18632/aging.102285)
Supplement: Supplementary Methods [file aging-11-102285-s002.pdf]

SUPPLEMENTARY METHODS

Primary hepatocyte isolation

Primary hepatocytes were isolated from the livers of male mice as previously described [1]. In brief, livers were perfused with Hank's Balanced Salt Solution (Invitrogen) containing 5 mM glucose supplemented with 0.5 mM EGTA and 25 mM HEPES (pH 7.4 at 37°C) using a CTP100 peristaltic pump (ThermoFisher). After exsanguination of the liver, perfusion was changed to DMEM (Sigma-Aldrich D5546) supplemented with 100 U/mL Penicillin and 0.1 mg/mL Streptomycin (Pen/Strep), 15 mM HEPES, and 100 U/mL of collagenase (Type IV, Worthington). Then cells were liberated and cell suspension was then filtered through a 70 µM cell strainer and centrifuged at 50 g x 2 min 3 times. Following centrifugation, cell pellets were resuspended in DMEM (Sigma-Aldrich D5796) supplemented with Pen/Strep, 5 mM HEPES, 10 nM dexamethasone, and seeded into 24-well plates or 24-well Seahorse plates precoated with collagen I (Sigma-Aldrich). One hour later the media was changed to DMEM (Sigma-Aldrich D5546) supplemented with Pen/Strep, 5 mM HEPES, 10 nM dexamethasone and 10% FBS. Media was changed 3 h later to serum-free DMEM (Sigma-Aldrich D5546) supplemented with Pen/Strep, 5 mM HEPES, 10 nM dexamethasone and cells were cultured overnight culture for various experiments.

Rectal temperature

Rectal temperature was measured using a Temperature control unit HB 101/2 (LSI letica Scientific Instruments).

T4 and α-GSU determinations

T4 levels were determined using the total T4 enzyme immunoassay test kit (MP Biomedicals, 07BC-1007), according to the manufacturer's instructions. α-GSU levels were determined using a commercially available kit following the manufacturer's instructions (Abbexa, abx 254594) [2].

Glycated haemoglobin

HbA1c levels were determined in blood samples using Hemoglobin A1c (HbA1c) Assay kit (CrystalChem, 80099), according to the manufacturer's protocol.

Energy intake determination

In order to determine the basal daily energy intake, the total amount of chow consumed per cage was monitored weekly during 3-4 weeks. In order to determine the fasting-induced energy intake, animals were fasted overnight and the amount of chow consumed by each animal was measured after 8 hours.

Metabolic and physical activity

Mouse metabolic rate was assessed by indirect calorimetry in a 16-chamber TSE Phenomaster system (TSE Systems GmbH, Bad Homburg, Germany). Mice were housed singly with water and food available ad libitum and maintained at ~22°C under a 12:12-h light-dark cycle (light period 0700-1900). All mice were individually caged three days before the onset of the experiment for acclimation. The concentrations of oxygen and carbon dioxide were monitored at the inlet and outlet of the sealed chambers to calculate oxygen consumption. Each chamber was measured for 30 s at 30-min intervals and data were recorded for 72 h total. Locomotor activity was monitored using an infrared photocell beam grid. Food and water intake was automatically monitored using built-in sensors in each cage.

Fecal lipid content

Lipid extraction from 1 g of dried feces was performed by chloroform-methanol as previously described [3]

Transaminases determination

Got and Gpt levels were measured in blood samples using Reflotron GPT (Alanine transaminase) (Cat# 10745138) and Reflotron GOT (Aspartate transaminase) (Cat# 100745120) strips in a Reflotron Plus (Roche).

Cholesterol and triglyceride determinations

Cholesterol, HDL and low density/very low-density lipoprotein (LDL/VLDL) in serum were determined using the EnzyChrom AF HDL and LDL/VLDL Assay kit (BioAssay Systems, E2HL-100), according to manufacturer's instructions. Triglyceride levels in tissues and plasma were determined using an EnzyChrom Triglyceride Assay Kit (BioAssay Systems, ETGA-200), according to manufacturer's instructions.

Lipidomic analyses

Lipids were extracted from approximately 20 mg of tissue using hexane-isopropanol as previously described [4] and the total lipid weight was determined

gravimetrically. Total oil, measured as fatty acids, fatty acids composition and triacylglycerol (TAG) species were analyzed by GLC as previously described [5]. In brief, fatty acid methyl esters (FAMES) were obtained from isolated lipids by heating the samples at 80 °C for 1 h in 3 ml of methanol/toluene/H2SO4 (88:10:2 v/v). Heptadecanoic acid (1/10 w/w) was added to each sample as an internal standard to allow quantification. After cooling, 1 ml of heptane was added and the samples were mixed. The FAMES were recovered from the upper phase and then separated and quantified using a Hewlett–Packard 5890A gas chromatograph (Palo Alto, CA, USA) with a Supelco SP-2380 capillary column of fused silica (30 m length, 0.25 mm i.d., 0.20 µm film thickness) (Bellefonte, PA, USA). Hydrogen was used as the carrier gas, with the linear gas rate being 28 cm/s. The detector and injector temperatures were set at 220 °C and the oven was set at 170 °C, with a split ratio was 1:50. Fatty acids were identified using standards (Sigma, St. Louis, MO, USA). TAGs were separated and quantified by GLC as previously described [6] with an Agilent 6890 gas chromatograph (Palo Alto, CA, USA), and hydrogen was used as the carrier gas. Triheptadecanoic acid was added to the samples as internal standard for quantification. The injector and detector temperatures both were 380 °C, the oven temperature was 345 °C, and a head pressure gradient from 70 to 120 kPa was applied, changing this last parameter depending on the column. The gas chromatography capillary column was a J & W Scientific DB-17HT (15 m length, 0.25 mm i.d., 0.15 µm film thickness) (Folsom, CA, USA), with a linear gas rate of 50 cm/s, the split ratio was 1:80, and the detector was a flame ionization detector (FID). The different TAG molecules were identified with respect to known samples, and the FID response was corrected. Polar lipids were analyzed and quantified by HPLC. Separation by HPLC was carried out in a Waters 2695 Module (Milford, MA) equipped with a Waters 2420 ELSD. The column used was a Lichrospher 100 Diol 254-4 (5 µm; Merck) applying a method based in a linear binary gradient of solvent mixtures containing different proportions of hexane, 2-propanol, acetic acid, water and trimethylamine. The flow was 1 ml/min, data were processed using Empower software, and the ELSD was regularly calibrated using commercial high-purity standards for each lipid.

Mitochondrial complexes enzymatic activities

Complex I to III and II to III activities were measured in liver and gastrocnemius lysates by the reduction of cytochrome c in the presence of NADH or succinate, respectively, as previously described [7]. Results were expressed in nmol/mg protein/min or corrected by citrate synthase activity in the same lysates.

Mitochondrial superoxide generation

Superoxide production was determined by the reduction of partially acetylated cytochrome *c* [8]. Results were expressed in nmol/mg protein/min or corrected by citrate synthase activity in the same lysates.

Enzymatic activities

Citrate synthase activity in gastrocnemius and liver lysates was measured as previously described [7]. Results were expressed in nmol/mg protein/min.
Superoxide dismutase (Sod; E.C.1.15.1.1) activity was determined at 37 ºC using the commercial kit Ransod (Randox, UK). This method employs the xanthine/ xanthine oxidase reaction to generate superoxide radicals which react with 2-(4-iodophenyl)-3-(4-nitrophe-nol)-5-phenyl tetrazolium chloride to form a red formazan dye that absorbs at 500 nm. The superoxide dismutase activity is then measured by the degree of inhibition of this reaction. The activity of the assay is defined as the amount of Sod that inhibits the rate of formazan dye formation by 50 %. The Sod calibrator included in the kit was used. Cu–Zn-Sod activity (Sod cytosolic; Sod Cyt) was differentiated from Mn-Sod (Sod mitochondrial; Sod mit) on the basis of its sensitivity to 3 mM sodium cyanide. Results were expressed in nmol/mg protein/min.
Catalase (E.C.1.11.1.6) activity was determined by incubating samples for 1 min at 37 ºC in 66 mM phosphate buffer solution, pH 7.4, and 65 µmol H_2O_2. The reaction was stopped with 32.4 mM ammonium molybdate and the molybdate– H_2O_2 complex was measured at 405 nm. Results were expressed in nmol/mg protein/min. Glutathione reductase (Grd; E.C.1.6.4.2) activity was measured using the commercial 'Glutathione Reductase' kit (Randox, UK). Grd catalyzes the reduction of Glutathione disulfide (*e.g.* oxidized glutathione; GSSG) in the presence of NADPH, which is oxidized to $NADP^+$. Results were expressed in nmol/mg protein/min. Glutathione peroxidase (Gpx; E.C.1.11.1.9) activity was determined at 37 ºC using the commercial kit 'Ransel' (Randox, UK). Gpx catalyzes the oxidation of reduced glutathione (GSH) by cumene hydroperoxide. In the presence of Gr and NADPH, GSSG is immediately converted to GSH with concomitant oxidation of NADPH to $NADP^+$. The decrease in absorbance at 340 nm is measured. Results were expressed in nmol/mg protein/min. Glutathione S transferase activity (Gst; E.C.2.5.1.18) was determined in a spectrophotometer at 340 nm by measuring the formation of the conjugated of glutathione and 1-chloro-2,4-dinitrobenzene. Results were expressed in nmol/mg protein/min. NADH-Coenzyme Q oxidoreductase activity (Nqo1; EC 1.6.99.2) was measured as described [9] by following

the decrease in NADH absorbance at 340 nm adapted to a Cobas Mira Autoanalyzer. The reaction mixture at a final volume of 200 µL contained 25 mM Tris·HCl (pH 7.5), 0.01% Tween 20, 0.7 mg/mL BSA (pH 7.4), 40 µM menadione, 5 µM FAD, 200 µM NADH, and tissue extract. Measurements were made at 25 s intervals over a time period of 10 min. Results were expressed in nmol/mg protein/min.

Barnes Maze

The method was performed according to a previously published protocol [10]. In brief, on the pre-training trial, mice were pre-trained to enter the escape box by guiding them to the escape box and remaining there for 2 min. The following day started the training trial (4 days). Mice were trained four trials per day and trials were separated by 15 min. Mice were free to explore the maze for 3 min or until they entered into the escape box. Once mice entered the box the buzzer was turned off and the mice were allowed to stay in the box for 1 min. The following day, subjects received a probe trial for 90 s to check the short-retention memory. During probe trial the escape box was removed. Primary latency to reach the target hole and total attempts were recorded. 7 days after the trial performed to check short-retention memory subjects received an additional the probe trial for 90 s to check long-term retention memory. Mice were not trained during the time period between probe trials.

Immunohistochemistry

Dissected tissues were fixed in 4% paraformaldehyde. Pancreatic sections (5 µm thick) were deparaffinized and rehydrated as previously described. For hematoxylin and eosin staining sections were immersed in hematoxylin (Merck) and eosin (Merck) for 4 and 2 minutes, respectively. For immunofluorescence, antigen retrieval was performed by heating in 0.01 M sodium citrate buffer (pH 6). After 1 h blocking at room temperature, sections were incubated overnight at 4°C with primary antibodies (Supplementary Table 3). Subsequently, slides were incubated with secondary antibodies (Supplementary Table 3) for 1 h at room temperature followed by DAPI-nuclear staining (Life Technologies,Carlsbad, USA).

REFERENCES

1. Zhang W, Sargis RM, Volden PA, Carmean CM, Sun XJ, Brady MJ. PCB 126 and other dioxin-like PCBs specifically suppress hepatic PEPCK expression via the aryl hydrocarbon receptor. PLoS One. 2012; 7:e37103. https://doi.org/10.1371/journal.pone.0037103 PMID:22615911
2. López-Noriega L, Cobo-Vuilleumier N, Narbona-Pérez ÁJ, Araujo-Garrido JL, Lorenzo PI, Mellado-Gil JM, Moreno JC, Gauthier BR, Martín-Montalvo A. Levothyroxine enhances glucose clearance and blunts the onset of experimental type 1 diabetes mellitus in mice. Br J Pharmacol. 2017; 174:3795–810. https://doi.org/10.1111/bph.13975 PMID:28800677
3. Kraus D, Yang Q, Kahn BB. Lipid Extraction from Mouse Feces. Bio Protoc. 2015; 5:e1375. https://doi.org/10.21769/BioProtoc.1375 PMID:27110587
4. Hara A, Radin NS. Lipid extraction of tissues with a low-toxicity solvent. Anal Biochem. 1978; 90:420–26. https://doi.org/10.1016/0003-2697(78)90046-5 PMID:727482
5. Martínez-Force E, Ruiz-López N, Garcés R. The determination of the asymmetrical stereochemical distribution of fatty acids in triacylglycerols. Anal Biochem. 2004; 334:175–82. https://doi.org/10.1016/j.ab.2004.07.019 PMID:15464966
6. Fernández-Moya V, Martínez-Force E, Garcés R. Identification of triacylglycerol species from high-saturated sunflower (Helianthus annuus) mutants. J Agric Food Chem. 2000; 48:764–69. https://doi.org/10.1021/jf9903861 PMID:10725146
7. Spinazzi M, Casarin A, Pertegato V, Salviati L, Angelini C. Assessment of mitochondrial respiratory chain enzymatic activities on tissues and cultured cells. Nat Protoc. 2012; 7:1235–46. https://doi.org/10.1038/nprot.2012.058 PMID:22653162
8. Martin-Montalvo A, Mercken EM, Mitchell SJ, Palacios HH, Mote PL, Scheibye-Knudsen M, Gomes AP, Ward TM, Minor RK, Blouin MJ, Schwab M, Pollak M, Zhang Y, et al. Metformin improves healthspan and lifespan in mice. Nat Commun. 2013; 4:2192. https://doi.org/10.1038/ncomms3192 PMID:23900241
9. Martín-Montañez E, Millon C, Boraldi F, Garcia-Guirado F, Pedraza C, Lara E, Santin LJ, Pavia J, Garcia-Fernandez M. IGF-II promotes neuroprotection and neuroplasticity recovery in a long-lasting model of oxidative damage induced by glucocorticoids. Redox Biol. 2017; 13:69–81. https://doi.org/10.1016/j.redox.2017.05.012 PMID:28575743
10. Patil SS, Sunyer B, Höger H, Lubec G. Evaluation of spatial memory of C57BL/6J and CD1 mice in the Barnes maze, the Multiple T-maze and in the Morris water maze. Behav Brain Res. 2009; 198:58–68. https://doi.org/10.1016/j.bbr.2008.10.029 PMID:19022298
