# Supplementary material for: Inadequate control of thyroid hormones sensitizes to hepatocarcinogenesis and unhealthy aging
Source: Aging (Albany NY). 2019 Sep 13;11(18):7746–79. doi: 10.18632/aging.102285 (PMC6781991; doi:10.18632/aging.102285)
Supplement: Supplementary Figures [file aging-11-102285-s003.pdf]

SUPPLEMENTARY FIGURES

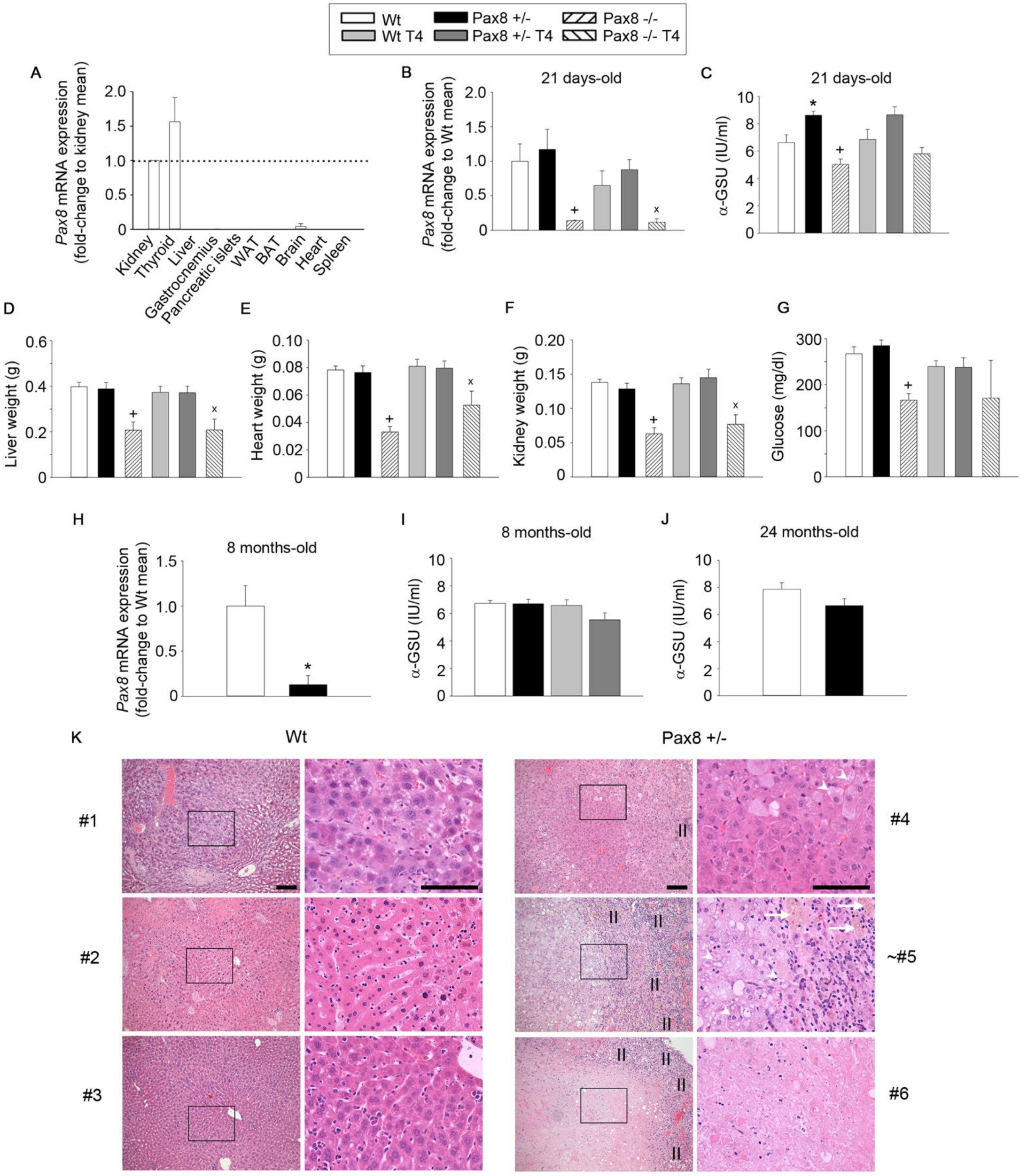

Supplementary Figure 1. Related to Figure 1. Characterization of murine models of severe hypothyroidism, mild hypothyroidism and hyperthyroidism. (**A**) Analysis of *Pax8* gene expression in different tissues in 8 month-old Wt mice. n = 3-6 kidney; n = 3 thyroid; n = 6 liver, n = 5 gastrocnemius; n = 3 pancreatic islets; n = 5 WAT; n = 6 BAT; n = 6 brain; n = 6 heart; n = 6 spleen. Reference line

indicates expression levels in kidney tissue. (**B**) Analysis of differential *Pax8* gene expression in thyroid tissue at 21 day of age. n = 7 Wt, n = 5 Pax8 +/-, n = 4 Pax8 -/-, n = 6 Wt T4, n = 5 Pax8 +/- T4, n = 4 Pax8 -/- T4. ANOVA on ranks. (**C**) Circulating α-GSU levels at 21 days of age. n = 6 Wt, n = 8 Pax8 +/-, n = 4 Pax8 -/-, n = 4 Wt T4, n = 4 Pax8 +/- T4, n = 4 Pax8 -/- T4. Two-way ANOVA. (**D**) Liver weight at 21 days of age. n = 21 Wt, n = 15 Pax8 +/-, n = 5 Pax8 -/-, n = 11 Wt T4, n = 11 Pax8 +/- T4, n = 4 Pax8 -/- T4. Two-way ANOVA. (**E**) Heart weight at 21 days of age. n = 21 Wt, n = 15 Pax8 +/-, n = 5 Pax8 -/-, n = 10 Wt T4, n = 11 Pax8 +/- T4, n = 4 Pax8 -/- T4. Two-way ANOVA. (**F**) Kidney weight at 21 days of age. n = 21 Wt, n = 14 Pax8 +/-, n = 5 Pax8 -/-, n = 11 Wt T4, n = 10 Pax8 +/- T4, n = 4 Pax8 -/- T4. Two-way ANOVA. (**G**) Blood glucose at 21 days of age. n = 14 Wt, n = 10 Pax8 +/-, n = 5 Pax8 -/-, n = 11 Wt T4, n = 11 Pax8 +/- T4, n = 4 Pax8 -/- T4. Two-way ANOVA. (**H**) Analysis of differential *Pax8* gene expression in thyroid tissue at 9 months of age. n = 3 per group. T-test two tailed. (**I**) Circulating α-GSU levels at 8 months of age. n = 7 per group. Two-way ANOVA. (**J**) Circulating α-GSU levels at 24 months of age. n = 7 per group. T-test two tailed. (**K**) Representative images of liver sections stained with hematoxylin and eosin at necropsies from Wt and Pax8+/- mice. Primary liver epithelial neoplasms were more frequently found in Pax8 +/- mice. II: inflammatory infiltrate; White arrow, cholestasis; White head arrow: steatosis. Scale bar 100 μm. #1: primary liver epithelial neoplasm. #2: normal liver. #3: normal liver. #4: primary liver epithelial neoplasm. #5: primary liver epithelial neoplasm. #6: primary liver epithelial neoplasm. n = 4 per group. Data are represented as the mean ± SEM. * p-value < 0.05 between Wt mice and Pax8 +/- mice. + p-value < 0.05 between Wt mice and Pax8 -/- mice. & p-value < 0.05 between Wt mice and Wt T4 mice. # p-value < 0.05 between Wt mice and Pax8 +/- T4 mice. x p-value < 0.05 between Wt mice and Pax8 -/- T4.

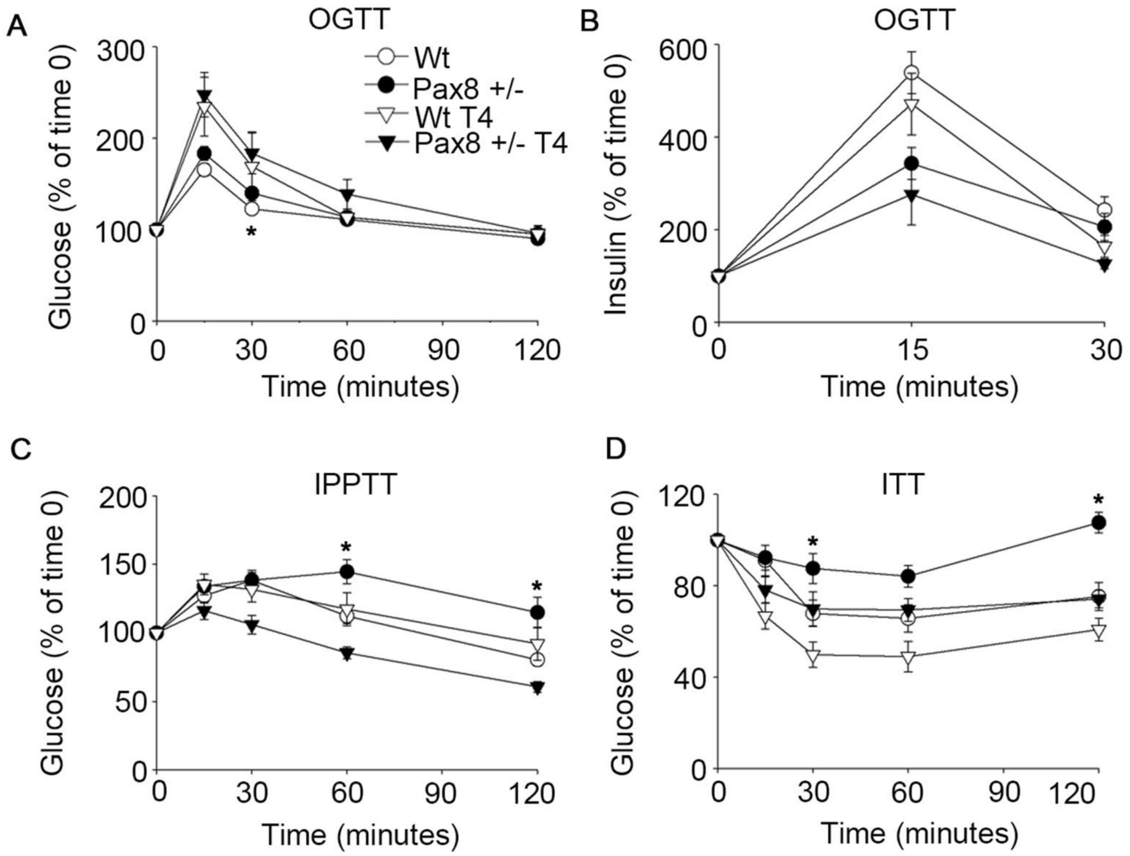

Supplementary Figure 2. Related to Figure 2. The modulation of THs alters glucose homeostasis and pancreatic islets in adulthood. (**A**) Glucose concentration in blood during the OGTT expressed as the percentage of basal glucose. n = 8 per group. (**B**) Circulating insulin during the OGTT expressed as the percentage of basal insulin. n = 8 per group. (**C**) Glucose concentration in blood during the IPPTT expressed as the percentage of basal glucose. n = 8 per group. (**D**) Glucose concentration in blood during the ITT expressed as the percentage of basal glucose. n = 8 per group. Data are represented as the mean ± SEM. * p-value < 0.05 between Wt mice and Pax8 +/- mice. & p-value < 0.05 between Wt mice and Wt T4 mice. # p-value < 0.05 between Wt mice and Pax8 +/- T4 mice. ANOVA on ranks.

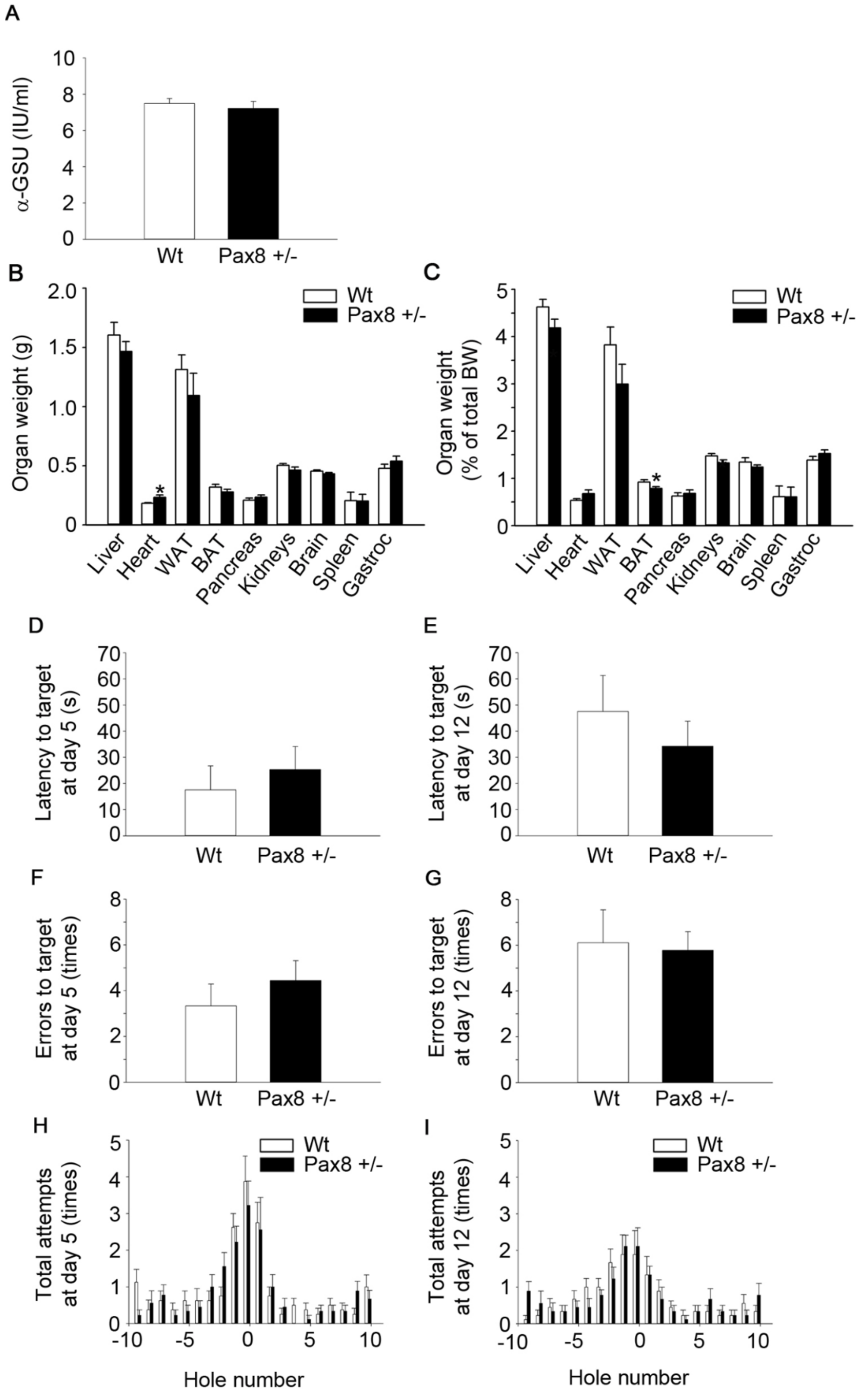

Supplementary Figure 3. Related to Figure 3. Mild hypothyroid Pax8 +/- mice fed a HFD do not exhibit major alterations on tissue weight and neurocognitive function. (**A**) Circulating α-GSU levels at 7 months of life. n = 7 Wt, n = 4 Pax8 +/-. (**B**) Tissues weight at sacrifice (9 months-old). n = 9 per group. (**C**) Tissues weight corrected by total body weight at sacrifice (9 months-old). n = 9 per group. (**D**) Latency to target at day 5 in Barnes maze. n = 9 per group. (**E**) Latency to target at day 12 in Barnes maze. n = 9 per group. (**F**) Errors to target at day 5 in Barnes maze. n = 9 per group. (**G**) Errors to target at day 12 in Barnes maze. n = 9 per group. (**H**) Total attempts to target in Barnes maze at day 5. n = 9 per group. (**I**) Total attempts to target in Barnes maze at day 12. n = 9 per group. Data are represented as the mean ± SEM. Barnes Maze experiments were performed at week 19-20 of HFD feeding (8 month–old). * p-value < 0.05. T-test two tailed.

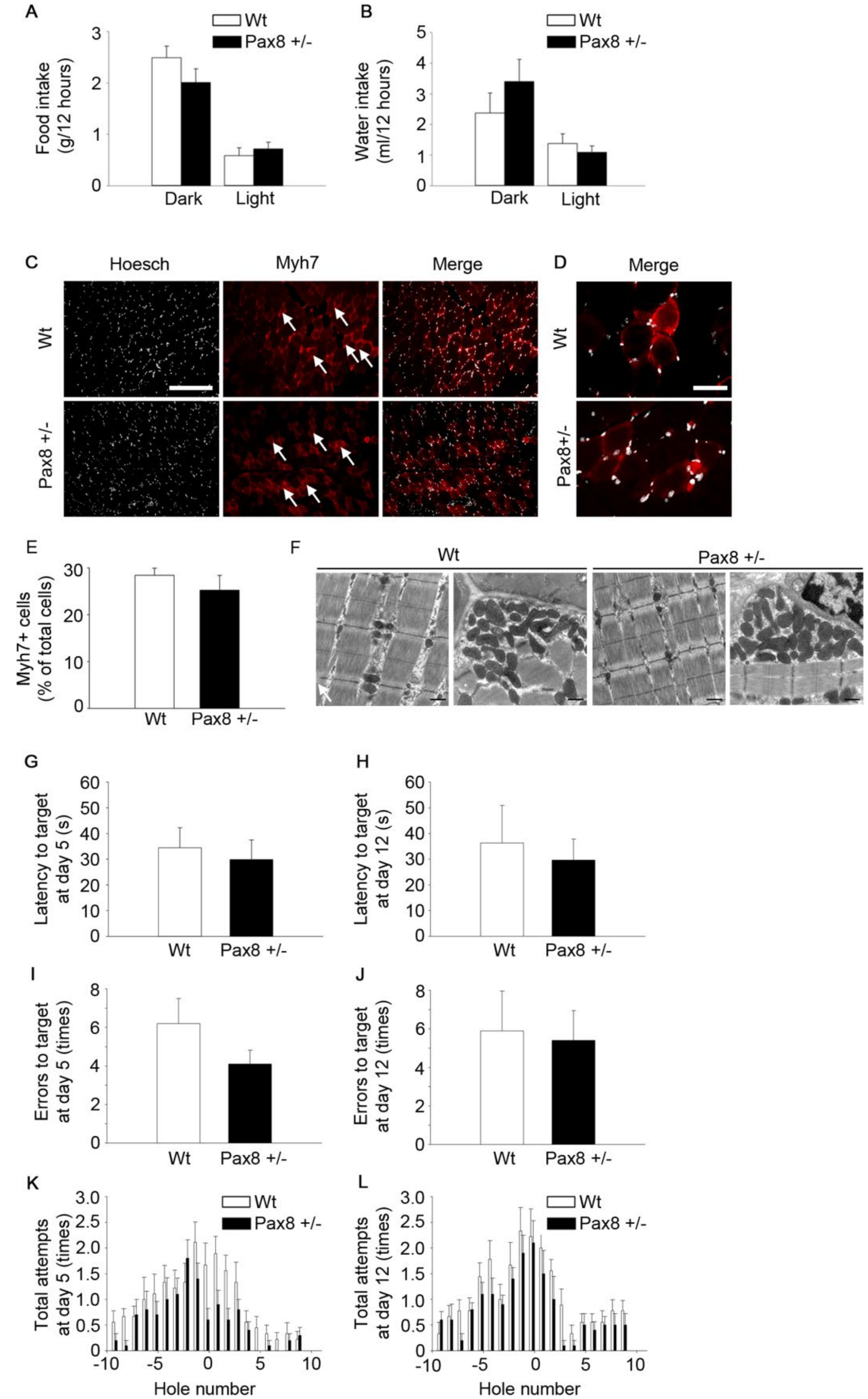

Supplementary Figure 4. Related to Figure 4. Mild hypothyroid Pax8 +/- mice fed a STD do not exhibit alterations food intake, water intake and neurocognitive function. (**A**) Energy intake determined during indirect calorimetry experiments. n = 8 Wt, n = 7 Pax8 +/-. (**B**) Water consumption determined during indirect calorimetry experiments. n = 8 Wt, n = 7 Pax8 +/-. (**C**) Myh7 immunostaining of gastrocnemius sections. Representative Myh7 expressing myofibers are marked with arrows. n = 6 per group. Scale bar: 250 µm. (**D**) Magnifications of Myh7 positive myofibers of gastrocnemius sections. n = 6 per group. Scale bar: 50 µm. (**E**) Quantification of the percentage of Myh7 expressing myofibers in the gastrocnemii. n = 6 per group. (**F**) Representative transmission electron microscopy images of gastrocnemius sections. n = 6 per group. Scale bar: 500 nm. (**G**) Latency to target at day 5 in Barnes maze. n = 10 per group. (**H**) Latency to

target at day 12 in Barnes maze. n = 10 per group. (**I**) Errors to target at day 5 in Barnes maze. n = 10 per group. (**J**) Errors to target at day 12 in Barnes maze. n = 10 per group. (**K**) Total attempts to target in Barnes maze at day 5. n = 10 per group. (**L**) Total attempts to target in Barnes maze at day 12. n = 10 per group. Mice were 7 month-old in indirect calorimetry experiments. Mice were 8 month-old in Barnes Maze experiments. Mice were 9 month-old at the time of killing. Data are represented as the mean ± SEM. * p-value < 0.05. T-test two tailed.

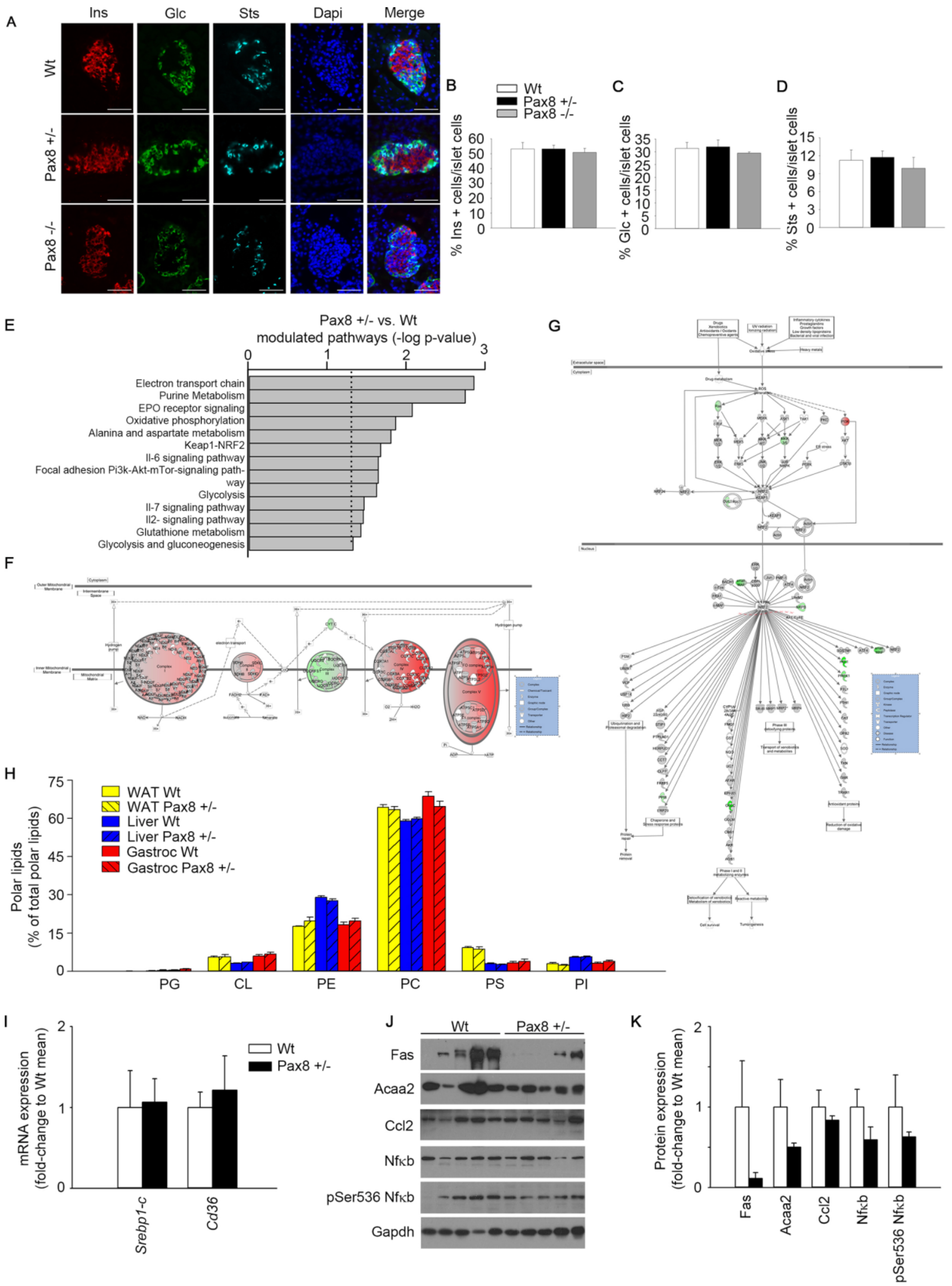

Supplementary Figure 5. Related to Figure 5. Mild hypothyroid Pax8 +/- mice exhibit metabolic alterations in metabolic tissues. (**A**) Representative immunofluorescence images of pancreatic sections from untreated Wt, Pax8 +/- and Pax8 -/- male mice at postnatal day 21. Scale bar: 50 µm. n = 4 per group. (**B**) Percentage of insulin positive cells in islet cells at postnatal day 21. n = 4 per group. One-way ANOVA. (**C**) Percentage of glucagon positive cells in islet cells at postnatal day 21. n = 4 per group. One-way ANOVA. (**D**) Percentage of somatostatin positive cells in islet cells at postnatal day 21. n = 4 per group One-way ANOVA. (**E**) Analysis of significantly modulated

pathways in pancreatic islets using Transcription Analysis Console platform. n = 3 per group. Statistical analysis was performed using Transcription Analysis Console using significantly modulated genes with a fold change ≥ 2.5 or a fold change ≤-2.5. (**F**) Representative picture of the annotated canonical pathway "oxidative phosphorylation" generated by Ingenuity Pathway Analysis platform. (**G**) Representative picture of the annotated canonical pathway "NRF2-mediated oxidative stress response" generated by Ingenuity Pathway Analysis platform. (**H**) Lipidomic analysis depicting percentages of the different species of polar lipids in WAT, liver and gastrocnemius. PG: Phosphatidylglycerol. CL: Cardiolipin. PE: Phosphatidylethanolamine. PC: Phosphatidylcholine PS: Phosphatidylserine PI: Phosphatidylinositol. n = 5 Wt, n = 6 Pax8 +/-. Two-way ANOVA. (**I**) mRNA expression of genes involved in lipid synthesis/import in gastrocnemius. *Srebp1-c*: n = 6 Wt, n = 5 Pax8 +/-; *Cd36*: n = 5 Wt, n = 6 Pax8 +/-. T-test two tailed. (**J**) Western blots showing proteins involved in lipogenesis in gastrocnemius lysates. n = 5 per group. (**K**) Densitometric analysis of western blots shown in panel J. T-test two tailed. Data are represented as the mean ± SEM. Ins: insulin. Glc: glucagon. Sts: somatostatin. Unless otherwise stated, mice were 9 month-old at the time of killing. * p-value < 0.05.

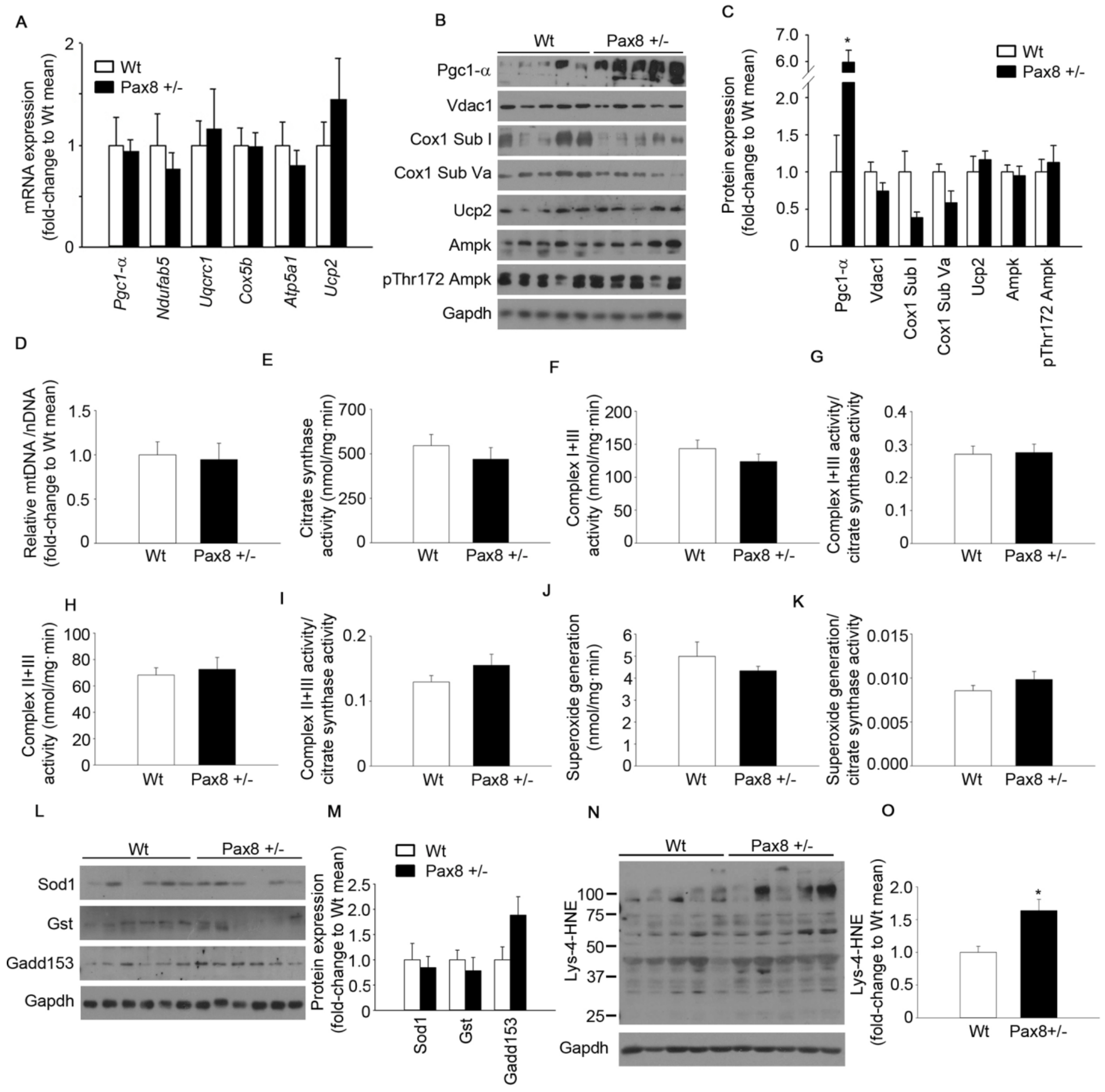

Supplementary Figure 6. Related to Figure 6. Mild hypothyroidism increases the accumulation of oxidative damage in the gastrocnemius. (**A**) mRNA expression of genes involved in mitochondrial biogenesis and function in the gastrocnemii. *Pgc1-α*: n = 5 Wt, n = 4 Pax8 +/-. *Ndufab5*: n = 5 Wt, n = 4 Pax8 +/-. *Uqcrc1*: n = 6 Wt, n = 5; Pax8 +/-. *Cox5b*: n = 5 Wt, n = 4 Pax8 +/-. *Atp5a1*: n = 5 Wt, n = 4 Pax8 +/-. *Ucp2*: n = 5 per group. (**B**) Western blots showing proteins involved in mitochondrial biogenesis/function as well as Ampk and its phosphorylated isoform in gastrocnemius lysates. n = 5 per group. (**C**) Densitometric analysis of western blots shown in panel B. n = 5 per group. (**D**) Relative mitochondrial DNA content in gastrocnemius isolations. n = 6 per group. (**E**) Citrate synthase activity in gastrocnemius

extracts. n = 6 per group. (**F**) Complex I+III activity in gastrocnemius extracts. n = 6 per group. (**G**) Complex I+III activity corrected by citrate synthase activity in gastrocnemius extracts. n = 6 per group. (**H**) Complex II+III activity in gastrocnemius extracts. n = 6 per group. (**I**) Complex II+III activity corrected by citrate synthase activity in gastrocnemius extracts. n = 6 per group. (**J**) Superoxide generation in mitochondrial complexes in gastrocnemius extracts. n = 5 Wt, n = 6 Pax8 +/-. (**K**) Superoxide generation in mitochondrial complexes corrected by citrate synthase activity in gastrocnemius extracts. n = 5 Wt, n = 6 Pax8 +/-. (**L**) Western blots showing expression levels of antioxidant and stress response proteins in gastrocnemius lysates. n = 6 per group. (**M**) Densitometric analysis of the western blot shown in panel L. (**N**) Western blot showing Lys-4-HNE staining in gastrocnemius extracts. n = 5 per group. (**O**) Densitometric analysis of western blots shown in panel N. Mice were 9 month-old at the time of killing. Data are represented as the mean ± SEM. * p-value < 0.05. T-test two tailed.
