# Supplementary material for: Inadequate control of thyroid hormones sensitizes to hepatocarcinogenesis and unhealthy aging
Source: Aging (Albany NY). 2019 Sep 13;11(18):7746–79. doi: 10.18632/aging.102285 (PMC6781991; doi:10.18632/aging.102285)
Supplement: Supplementary Tables [file aging-11-102285-s001.pdf]

# SUPPLEMENTARY TABLES

**Supplementary Table 1. Major gross pathologies identified at necropsy.**

| Tissue | Pathology | Wt (52/ %) | Wt T4 (14/ %) | Pax8 +/- (17/ %) | Pax8 +/- T4 (18/ %) | Pax8 -/- (8/ %) | Pax8 -/- T4 (5/%) |
|---|---|---|---|---|---|---|---|
| Heart | Enlarged | 4/ 8% | 1/ 7% | 1/ 7% | 4/ 22% | 0/ 0% | 0/ 0% |
| Kidney | Necrotic | 4/ 8% | 0/ 0% | 1/ 7% | 0/ 0% | 0/ 0% | 0/ 0% |
| Liver | Cancer | 9/ 17% | 0/ 0% | 8/ 47%* | 0/ 0% | 0/ 0% | 0/ 0% |
| | Enlarged | 14/ 27% | 0/ 0% | 9/ 53% | 2/ 11% | 0/ 0% | 0/ 0% |
| Spleen | Enlarged | 9/ 17% | 0/ 0% | 7/ 41% | 3/ 17% | 0/ 0% | 0/ 0% |

The percentage of mice presenting with various pathologies is represented. * $p < 0.05$ compared to Wt. Analysed using Fisher's Exact Test.

**Supplementary Table 2. List of the most modulated transcripts in pancreatic islets isolated from Pax8 +/- and Wt mice.**

| Gene Symbol | Fold Change | p-value |
|---|---|---|
| *Scgn* | 30.08 | 0.0253 |
| *Gm11096* | 28.36 | 0.0035 |
| *Pcsk1n* | 15.77 | 0.0450 |
| *Slc2a5* | 12.74 | 0.0440 |
| *Mlxipl* | 12.64 | 0.0461 |
| *Wdr83os* | 12.49 | 0.0310 |
| *Abat* | 12.43 | 0.0202 |
| *Slc25a42* | 11.52 | 0.0277 |
| *Insrr* | 11.46 | 0.0420 |
| *Cfap126* | 11.24 | 0.0204 |
| *Serpini2* | −10.51 | 0.0278 |
| *Galnt3* | −10.7 | 0.0335 |
| *Ero1l* | −10.99 | 0.0183 |
| *Tm4sf1* | −13.3 | 0.0471 |
| *Igfbp3* | −16.46 | 0.0464 |
| *Ak4* | −21.78 | 0.0363 |
| *Hmox1* | −24.1 | 0.0341 |
| *Reg2* | −29.21 | 0.0465 |
| *Pfkfb3* | −31.12 | 0.0393 |
| *Slc2a1* | −42.5 | 0.0461 |

**Supplementary Table 3. Antibodies used in this study.**

| Antibody | Dilution | Vendor | Catalog number |
|---|---|---|---|
| Alexa fluor 647 donkey anti-mouse | 1:800 | Thermofisher | Cat# A-31571, RRID:AB_162542): |
| Alexa fluor 555 donkey anti-rabbit | 1:800 | Thermofisher | Cat# A-31572, RRID:AB_162543 |
| Alexa fluor 488 donkey anti-goat | 1:800 | Thermofisher | Cat# A-11055, RRID:AB_2534102 |
| Anti-insulin | 1:500 | Sigma-Aldrich | Cat# I2018, RRID:AB_260137 |
| Anti-insulin | 1:500 | Santa Cruz Biotechnology | Cat# sc-9168, RRID:AB_2126540 |
| Anti-glucagon | 1:150 | Cell Signaling | Cat# 2760, RRID:AB_659831 |
| Anti-glucagon | 1:100 | Sigma-Aldrich | Cat# G2654, RRID:AB_259852 |
| Anti-somatostatin | 1:150 | Santa Cruz Biotechnology | Cat# sc-7819, RRID:AB_2302603 |
| Anti-pSer473 Akt | 1:500 | Cell Signaling | Cat# 9271, RRID:AB_329825 |
| Anti-Akt | 1:1000 | Cell Signaling | Cat# 9272, RRID:AB_329827 |
| Anti-Gapdh | 1:1000 | Cell Signaling | Cat# 2118, RRID:AB_561053 |
| Anti-pThr172 Ampk | 1:1000 | Cell Signaling | Cat# 2535, RRID:AB_331250 |
| Anti-Ampk | 1:1000 | Cell Signaling | Cat# 2532, RRID:AB_330331 |
| Anti-Lc3b | 1:1000 | Cell Signaling | Cat# 2775, RRID:AB_915950) |
| Anti-Ucp2 | 1:200 | Santa Cruz Biotechnology | Cat# sc-6526, RRID:AB_2213582 |
| Anti-Vdac1 | 1:1000 | Abcam | Cat# ab15895, RRID:AB_2214787 |
| Anti-Pgc1-α | 1:1000 | Santa Cruz Biotechnology | Cat# sc-517380, RRID:AB_2755043 |
| Anti- cytochrome *c* oxidase subunit Va | 1:1000 | Molecular probes | Cat# A21363, RRID:AB_1501848 |
| Anti- cytochrome *c* oxidase subunit I | 1:1000 | Molecular probes | Cat# A-6403, RRID:AB_221582 |
| Anti-4hne | 1:1000 | Millipore | Cat# 393206-100UL, RRID:AB_211975 |
| Anti-Fas | 1:200 | Santa Cruz Biotechnology | Cat# sc-55580, RRID:AB_2231427 |
| Anti-Acaa2 | 1:200 | Santa Cruz Biotechnology | Cat# sc-100847, RRID:AB_2219392 |
| Anti-Ccl2 | 1:200 | ENZO | Cat# ALX-804-549-C100, RRID:AB_2050978 |
| Anti-NfκB | 1:1000 | Abcam | Cat# ab32536, RRID:AB_776751 |
| Anti-pSer536 NfκB | 1:1000 | Cell Signaling | Cat# 3033, RRID:AB_331284 |
| Anti-Sod1 | 1:2000 | Abcam | Cat# ab13498, RRID:AB_300402 |
| Anti-Gst | 1:1000 | Sigma-Aldrich | Cat# G1160, RRID:AB_259845 |
| Anti-Gadd153 | 1:200 | Santa Cruz Biotechnology | Cat# sc-575, RRID:AB_631365 |
| Anti-Myh7 | 1:150 | Santa Cruz Biotechnology | Cat# sc-53090, RRID:AB_2147279 |
| HRP-goat anti-rabbit | 1:5000 | Sigma-Aldrich | Cat# A0545, RRID:AB_257896 |
| HRP-rabbit anti-mouse | 1:5000 | Sigma-Aldrich | Cat# A9044, RRID:AB_258431 |
| HRP-IgGκ light chain binding protein anti-mouse | 1:1000 | Santa Cruz Biotechnology | Cat# sc-516102, RRID:AB_2687626 |
| HRP-donkey anti-goat | 1:2000 | Jackson ImmunoResearch Labs | Cat# 705-036-147, RRID:AB_2340392 |

**Supplementary Table 4. Primers used for RT-PCR in this study.**

| Gene | Forward primer sequence | Reverse primer sequence |
|---|---|---|
| Mouse mtNd1 | 5′–CCTATCACCCTTGCCATCAT–3′ | 5′–GAGGCTGTTGCTTGTGTGAC–3′ |
| Mouse nPecam | 5′–ATGGAAAGCCTGCCATCATG–3′ | 5′–TCCTTGTTGTTCAGCATCAC–3′ |
| Mouse Pgc1-α | 5′–GGGTCAGAGGAAGAGATAAAGTTG–3′ | 5′–CACCAAACCCACAGAAAACAG–3′ |
| Mouse Pax8 | 5′–GTTTGAGCGGCAGCATTAC–3′ | 5′–GTAAGGGCAGTGGGTACAGC–3′ |
| Mouse Rps29 | 5′-GGAGTCACCCACGGAAGTT-3′ | 5′-CATGTTCAGCCCGTATTTGC-3′ |
| Mouse Ucp2 | 5′-GCTTGGGATCCTGGAACGT-3′ | 5′-GGCAGCCATTAGGGCTCTTT-3′ |
| Mouse Srebp1-c | 5′-GCATGCCATGGGCAAGTAC-3′ | 5′–AGCATCTCCTGCGCACTCA–3′ |
| Mouse Srebp2 | 5′- ACCGGTCCTCCATCAACG-3′ | 5′- CCAGGTCGATGCCCTTCA-3′ |
| Mouse Fasn | 5′-TCCTGAATCAGCCCACG-3′ | 5′-ACACCCATGAGCGAGTCC-3′ |
| Mouse Cd36 | 5′-CACAGACGCAGCCTCCTT-3′ | 5′-TGGATTCTGGAGGGGTGA-3′ |
| Mouse Socs2 | 5′-GTAAGGGCAGTGGGTACAGC-3′ | 5′-GGTAAAGGGAGTCCCCAGA-3′ |
| Mouse Il-1β | 5′-AACTGTTGGTGAGGAATGTGG-3′ | 5′-GGTCCTGTCCCTCTTGTTTTCA-3′ |
| Mouse Tnf-α | 5′-CCCTCACACTCAGATCATCTTCT-3′ | 5′-GCTACGACGTGGGCTACAG-3′ |
| Mouse Cpt1 | 5′-AGACAAGAACCCCAACATCC-3′ | 5′-CAAAGGTGTCAAATGGGAAGG-3′ |
| Mouse Lcad | 5′-GGTGGAAAACGGAATGAAAGG-3′ | 5′-GGCAATCGGACATCTTCAAG-3′ |
| Mouse Mcad | 5′-TGTTAATCGGTGAAGGAGCAG-3′ | 5′-CTATCCAGGGCATACTTCGTG-3′ |
| Mouse Atp5a1 | 5′-CATTGGTGATGGTATTGCGC-3′ | 5′-TCCCAAACACGACAACTCC-3′ |
| Mouse Cox5b | 5′-ACCCTAATCTAGTCCCGTCC-3′ | 5′–CAGCCAAAACCAGATGACAG–3′ |
| Mouse Uqcrc1 | 5′-ATCAAGGCACTGTCCAAGG-3′ | 5′-TCATTTTCCTGCATCTCCCG-3′ |
| Mouse Ndufab5 | 5′-GGACCGAGTTCTGTATGTCTTG-3′ | 5′-AAACCCAAATTCGTCTTCCATG-3′ |
| Mouse Cish | 5′–GCATAGCCAAGACGTTCTCC–3′ | 5′–AATGTACCCTCCGGCATCTT–3′ |
